# BET bromodomain inhibition rescues PD-1-mediated T-cell exhaustion in acute myeloid leukemia

**DOI:** 10.1038/s41419-022-05123-x

**Published:** 2022-08-02

**Authors:** Mengjun Zhong, Rili Gao, Ruocong Zhao, Youxue Huang, Cunte Chen, Kehan Li, Xibao Yu, Dingrui Nie, Zheng Chen, Xin Liu, Zhuandi Liu, Shaohua Chen, Yuhong Lu, Zhi Yu, Liang Wang, Peng Li, Chengwu Zeng, Yangqiu Li

**Affiliations:** 1 Key Laboratory for Regenerative Medicine of Ministry of Education, Institute of Hematology, Jinan University, 510632 Guangzhou, P. R. China; 2 Center for Cell Regeneration and Biotherapy, Guangzhou Institutes of Biomedicine and Health, Chinese Academy of Sciences, 510530 Guangzhou, P. R. China; 3 Department of Hematology, First Affiliated Hospital, Jinan University, 510632 Guangzhou, P. R. China; 4 Department of Oncology, First Affiliated Hospital, Jinan University, 510632 Guangzhou, P. R. China

**Keywords:** Acute myeloid leukaemia, Preclinical research

## Abstract

Sustained expression of programmed cell death receptor-1 (PD-1) is correlated with the exhaustion of T cells, and blockade of the PD-1 pathway is an effective immunotherapeutic strategy for treating various cancers. However, response rates are limited, and many patients do not achieve durable responses. Thus, it is important to seek additional strategies that can improve anticancer immunity. Here, we report that the bromodomain and extraterminal domain (BET) inhibitor JQ1 inhibits PD-1 expression in Jurkat T cells, primary T cells, and T-cell exhaustion models. Furthermore, JQ1 dramatically impaired the expression of PD-1 and T-cell immunoglobulin mucin-domain-containing-3 (Tim-3) and promoted the secretion of cytokines in T cells from patients with acute myeloid leukemia (AML). In line with that, BET inhibitor-treated CD19-CAR T and CD123-CAR T cells have enhanced anti-leukemia potency and resistant to exhaustion. Mechanistically, BRD4 binds to the NFAT2 and PDCD1 (encoding PD-1) promoters, and NFAT2 binds to the PDCD1 and HAVCR2 (encoding Tim-3) promoters. JQ1-treated T cells showed downregulated NFAT2, PD-1, and Tim-3 expression. In addition, BET inhibitor suppressed programmed death-ligand 1 (PD-L1) expression and cell growth in AML cell lines and in primary AML cells. We also demonstrated that JQ1 treatment led to inhibition of leukemia progression, reduced T-cell PD-1/Tim-3 expression, and prolonged survival in MLL-AF9 AML mouse model and Nalm6 (B-cell acute lymphoblastic leukemia cell)-bearing mouse leukemia model. Taken together, BET inhibition improved anti-leukemia immunity by regulating PD-1/PD-L1 expression, and also directly suppressed AML cells, which provides novel insights on the multiple effects of BET inhibition for cancer therapy.

## Introduction

Cancer cells can evade T-cell immune responses by driving T-cell exhaustion [1]. Exhausted T cells are characterized by the excessive expression of multiple inhibitory receptors or immune checkpoint molecules, including programmed cell death receptor-1 (PD-1), cytotoxic T lymphocyte-associated molecule-4 (CTLA-4), and T-cell immunoglobulin mucin-domain-containing-3 (Tim-3) [2, 3]. Numerous studies highlight the inhibitory receptor PD-1 as a key driver in the process of T-cell exhaustion [4, 5]. The interaction between PD-1 and its ligands (PD-L1 and PD-L2) has been shown to restrain T-cell activity [6, 7]. Our previous findings have shown that the percentage of PD-1 + T cells are increased in patients with hematologic malignancies [8, 9], and increased expression of PD-1 in T cells was reported as an independent adverse risk factor for treatment response and survival in acute myeloid leukemia (AML) [10]. T cells from AML patients display a signature of T-cell exhaustion which is more prominent in the bone marrow, similar to exhaustion signatures of solid cancers [11]. Cancer immunotherapies based on reinvigorating exhausted T cells, such as PD-1 blockade, have demonstrated therapeutic prospects in a number of solid tumors [12–14]. Recently, several clinical trials of PD-1, PD-L1, CTLA-4, and Tim-3 blockade have been performed in patients with hematological malignancies [15, 16]. However, unlike the effects of immune checkpoint blockade in solid tumors [17, 18], a single blockade of one inhibitory pathway does not achieve a durable response for hematological malignancies, particularly for AML [19–21]. AML may use a variety of inhibitory pathways to evade T-cell immune attack, and inhibition of one inhibitory receptor may cause upregulation of others [22, 23]. It remains a great challenge to seek additional strategies that can induce more robust and durable T-cell immune responses.

The clinical success of PD-1 blockade highlights the importance of further investigation of the mechanisms by which PD-1 is regulated in T-cell activation and exhaustion. PD-1 is dynamically regulated by multiple pathways and plays a pivotal role in regulating T-cell exhaustion. However, some evidence has been obtained indicating that exhausted T cells that were reinvigorated by PD-1 blockade became “reexhausted” when the tumor antigen concentration remains high [24]. Previous studies have also shown that the expression of exhaustion-associated genes is reinforced, resulting in stable maintenance of the exhaustion-associated characteristics of exhausted T cells [25]. These observations suggest that PD-1/PD-L1 pathway blockade can reverse the exhausted state but cannot fully reprogram exhausted T cells. T-cell exhaustion epigenetic or transcriptional programs are maintained, which might restrict cellular responses during PD-1 blockade therapy. These studies have generated substantial interest in identifying the upstream PD-1-regulatory pathway that may contribute to the establishment and maintenance of T-cell exhaustion. Therefore, it is crucial to define combinatorial approaches that reduce the expression of PD-1 and reverse T-cell exhaustion epigenetic programs. Many transcription factors (TFs), including Thymocyte selection-associated HMG box protein (TOX) [26, 27], nuclear factor of activated T cells (NFAT) [28], activator protein 1 (AP-1) [29], nuclear factor kappa-B (NF-κB) [30], and signal transducer and activator of transcription (STAT) family members [31, 32], have been demonstrated to regulate PD-1 expression, and these studies have implications for therapeutic opportunities. Here, we sought to identify small-molecule inhibitors that target PD-1 upstream regulators and improve anticancer T-cell responses.

The bromodomain and extraterminal domain (BET) protein family contains BRDT, BRD2, BRD3, and BRD4. BRD4 interacts with multiple protein complexes in the active promoters and enhancers, functions as a general regulator of RNA polymerase II (Pol II)-dependent transcription, and regulates the expression of some essential genes [33–35]. Several structure/activity-based BET protein inhibitors have been developed, and the anticancer activity of BET inhibitors has been demonstrated in preclinical models and clinical trials [36–38]. Recently published studies further show that BET inhibitors promote anticancer immunity via suppressing PD-L1 expression by reducing the binding of BRD4 to its promoter. BET inhibitors such as JQ1 have also improved T-cell-mediated antitumor immunity [39, 40]. However, the effect and mechanisms of BET inhibitors in T-cell exhaustion remain largely unknown. In this study, we demonstrated that BET bromodomain inhibition suppressed PD-1 and Tim-3 expression via BRD4 and NFAT2, and augmented the cytokine production of T cells from AML patients, as well as rescued exhausted T cells via reducing the expression of PD-1 and Tim-3. BET inhibitors also prolonged the survival time of leukemia mice. Besides, BET inhibition suppressed PD-L1 expression and the growth of leukemia cell in AML. These findings might provide evidence and rationale that BET bromodomain inhibition enables therapy of AML by a dual action on exhausted T cells and AML cells.

## Materials and methods

### Clinical samples

A total of 29 bone marrow (BM) samples of newly diagnosed AML were collected from the Department of Hematology, First Affiliated Hospital of Jinan University, Guangzhou, Guangdong, China (Supplemental Table 1). All healthy donors and AML patients were informed about the study and provided consent prior to blood collection. Ethical approval for this research (No. 20200326) was obtained from the Ethics Committee of the First Affiliated Hospital of Jinan University.

### Cultured cells and CAR T cells production

Jurkat T cells, GFP/luciferase-expressing Nalm6 (Nalm6-GL) cells, THP1 cells, NB4 cells, HEL cells, and GFP/luciferase-expressing MV411 (MV411-GL) cells were cultivated in RPMI 1640 (Invitrogen) supplemented with 10% fetal calf serum (FBS, Gibco) at 37 °C in a humidified atmosphere with 5% carbon dioxide (CO_2_).

Mononuclear cells were isolated from BM or umbilical cord blood of collected samples using Ficoll density gradient. Primary T cells were positively selected by human CD4 microbeads, CD8 microbeads, or CD3 microbeads (Miltenyi Biotec) according to the manufacturer’s instructions, followed by stimulation with Transact^TM^ CD3/CD28 T-cell activator (MACS) and cultured in GT-T551 medium (Takara, Japan) supplemented with 5% fetal calf serum (Gibco), 1×BIOMYC-3 (FDbio Science), and 10 ng/ml IL-2. The purity of the T cells was >90% as measured by flow cytometric analysis.

Construction of the high-affinity anti-GD2 chimeric antigen receptor (HA-28z-CAR) plasmid was as described by Rachel C. Lynn et al. [41]. Anti-CD19 and anti-CD123 chimeric antigen receptor (CD19-CAR and CD123-CAR) plasmid were from Prof. Peng Li, and the construction of CD19-CAR T cell was followed as described by Prof. Peng Li [42]. Two days after activation with CD3/CD28 T-cell activator, primary T cells were transduced with HA-28z-CAR or GFP or CD19-CAR or CD123-CAR lentiviral vector. Medium was replaced with GT-T551 medium containing 5% FBS and 10 ng/ml IL-2 24 hours after infection, and T cells expanded for 5–10 days.

### Flow cytometric analysis

Cells were stained for flow cytometry in staining buffer (BD Pharmingen^TM^) with the indicated Abs at 4 °C for 30 min. Jurkat T cells were labeled with PE mouse anti-human CD279 (560795, EH12.1, BD Biosciences), and primary CD4 + and CD8 + T cells were stained with PE mouse anti-human CD279 and PE-Cy^TM^7 mouse anti-human CD69 (557745, FN50, BD Biosciences), which were detected by Beckman Coulter CytoFLEX (Suzhou, China). CD3 + T cells from AML patients were stained with CD4-FITC (555346, BD Biosciences), CD8-APC/Cy7 (344714, Biolegend), CD25-APC (555434, BD Biosciences), CD127-BV510 (563086, BD Biosciences), CD45RA-PerCP/Cy5.5 (304122, Biolegend), CCR7-BV421 (562555, BD Biosciences), PD-1-PE/Cy7 (561272, BD Biosciences), and Tim-3-PE (563422, BD Biosciences). A total of 30,000 CD3 + T cells were collected with BD Verse flow cytometer (BD, Bioscience, USA). All data analysis was performed with Flowjo software (Flowjo LLC, USA). Murine peripheral blood cells were stained with CD3-APC/Cy7 (100329, Biolegend), PD-1-APC (109112, Biolegend), and Tim-3-PE (134003, Biolegend).

For intracellular cytokine analysis, CD4 + and CD8 + T cells from healthy donors, and CD3 + T cells from AML patients were stimulated for 6 hours with P/I (PMA: 20 ng/L, ionomycin: 0.5 μM) in the presence of brefeldin A to allow cellular cytokine accumulation. Then, cells were fixed, permeabilized, and stained with IL-2-FITC (500305, Biolegend), IFN-γ-PE (506507, Biolegend), and TNF-α-APC (502913, Biolegend). Flow cytometry was used to evaluate the secretion of intracellular cytokines.

For analysis of apoptosis of T cells and cancer cells, the Annexin V-PE/7-AAD-PerCP/Cy5.5 apoptosis Kit (Multi science, China) and Annexin V-APC/PI apoptosis Kit ((Multi science, China) were used following the manufacturer’s instructions, respectively. Cells were collected by CytoFLEX, and data were analyzed by Flowjo software.

### Cell proliferation assays

The relative cell growth was quantified using Cell Counting Kit-8 (CCK-8, Dojindo, Tokyo, Japan). Briefly, 100 μl cells from different intervention groups were seeded onto triplicate wells of a 96-well plate at a concentration of 10,000 cells/well (for tumor cells) or 50,000 cells/well (for T cells), and 10 μl CCK-8 reagent was added to each well and incubated for 4 hours in indicated time point. The optical density (OD) was measured at 450 nm with a microplate reader (BioTek-Synergy4, USA).

### RNA extraction and quantitative real-time RT-PCR

Total RNA from Jurkat or CD8 + T cells was extracted with TRIzol reagent (Invitrogen) according to the manufacturer’s instructions. RNA was reverse transcribed into cDNA using the High-Capacity cDNA Reverse Transcription Kit. Real-time PCR was performed with SYBR Green, and the qRT-PCR cycling program was as follows: 40 cycles of 95 °C for 30 s, 60 °C for 30 s, and 72 °C for 30 s. Relative expression levels were calculated using the comparative ΔΔCt method, and data were normalized to β-actin mRNA levels. The primers used for real-time PCR analysis are listed in Supplemental Table 2a.

### Immunofluorescence

Cells were fixed in 4% formaldehyde prior to cell permeabilization with 0.1% Triton X-100. After washing with PBS, cells were blocked with Immunol Staining Blocking Buffer (Beyotime). Then, cells were incubated with mouse anti-NFAT2 antibody (1:20, ab2796 Abcam) overnight and then incubated with DyLight-488 conjugated secondary antibody (1:100, A23210, Abbkine). Nuclei were stained with DAPI (1:1000, 564907, BD Pharmingen^TM^), and images were taken with a Leica SP8 confocal microscope (Leica Corp, Germany).

### siRNA interference

Jurkat T cells were transfected using the Neon^®^ Transfection System (Invitrogen) with 100 pmol of oligonucleotides in 10 μl reactions [43–45]. Briefly, 2 × 10^5^ cells were suspended in 100 pmol of oligonucleotides in 10 μl reactions, and each 12-well plate was transfected four times. After electroporation, cells were cultured in 1640 medium containing 10% FBS at 37 °C with 5% CO_2_ for 48 hours, and were then divided into two groups: with and without 150 ng/ml PHA treatment for 24 hours, followed by harvested to examine the mRNA expression. siRNA sequences targeting NFAT2 and BRD4 are listed in Supplemental Table 3.

### Lentivirus transduction

A lentivirus overexpressing BRD4 (lv-BRD4) and a control lentivirus (lv-NC) was purchased commercially from Genechem (Shanghai, China). Jurkat T cells were transduced with lv-BRD4 or lv-NC lentivirus, and positively transduced cells were selected by 5 μg/mL puromycin for 5 days.

### Tumor-killing assay

Nalm6-GL target cells were incubated with CD19-CAR T cells at the E:T ratio of 1:1 in the six-well plate and target cell viability was monitored 72 hours later. Then CD19-CAR T cells were treated with DMSO or the indicated concentration of JQ1 for 72 hours after centrifugation, and the expression of PD-1 and Tim-3 and the proportion of CAR T cells were detected by FACS. After centrifugation, treated CD19-CAR T cells were co-cultured with Nalm6-GL cells at the E:T ratio of 1:1 in triplicate wells of 96-well plates. Target cell viability was monitored 48 hours later by adding 100 µl/well of D-luciferin (potassium salt) (Cayman Chemical, USA) at 150 µg/ml. Background luminescence was negligible (<1% of the signal from wells containing only target cells). The viability (%) was calculated as the experimental signal/maximal signal × 100, and the percentage of killing was equal to 100% − percent viability. The co-cultured supernatant of CD19-CAR T cells was collected for detecting the secretion of cytokines.

Similar to CD19-CAR T cells, CD123-CAR T cells were co-cultured with MV411-GL cells followed by being treated with the indicated concentration of JQ1 for 72 hours, and being co-cultured with MV411-GL cells again, the percentages of PD-1 and Tim-3 were detected by FACS, and the viability of MV411-GL cells was determined by a microplate reader. The co-cultured supernatant of CD123-CAR T cells was collected for detecting the secretion of cytokines.

### Enzyme-linked immunosorbent assay (ELISA)

ELISA kits for IL-2, IFN-γ, granzyme B, and TNF-α were purchased from DAKEWE (Shenzhen, China), and all ELISAs were performed according to the manufacturer’s protocols. The culture supernatants were collected and centrifuged at 500×*g* for 5 min, and the supernatants were subjected to the protocols provided by the manufacturer.

### Western blot analysis

Cells were harvested and lysed in RIPA buffer containing protease inhibitors. The cell lysates were run in a 10% SDS–PAGE Criterion X-gel (Bio-Rad) and then transferred to a polyvinylidene fluoride membrane. The membranes were blocked with QuickBlock™ Blocking Buffer (Beyotime, China) and probed with a primary antibody directed against NFAT2 (1:1000, Abcam, ab2796) and BRD4 (1:1000, Abcam, ab243862) overnight at 4 °C. Anti-β-actin antibody (1:5000, Thermo Fisher Scientific) served as control. After incubated with secondary antibody, signals from bound antibodies were amplified with ECL (Beyotime, China), and images were acquired in a UVITEC photo documenter. The full gel images for those cropped in the paper figures are shown in Supplemental Fig. 1.

### Chromatin immunoprecipitation (ChIP) assay

Chromatin immunoprecipitation (ChIP) assays were performed with anti-BRD4 (Abcam, ab243682), anti-NFAT2 (Abcam, ab2796), or non-immune mouse IgG control antibodies using EZ-ChIP™# 17-371 (Millipore, USA) according to manufacturer’s instructions, and ChIP-qPCR primers are listed in Supplemental Table 2b.

### RNA-seq analysis

Total RNA was extracted and examined for RIN number to inspect the RNA integrity. cDNA library construction and sequencing were performed by Shanghai Biotechnology Corporation with VAHTS Stranded mRNA-seq Library Prep Kit for Illumina^®^. Hisat2 (version:2.0.4) was used for the alignment of high-quality reads and mapping to the human reference genome (GRCh38). The expression value of each gene was calculated in terms of fragments per kilobase of exon model per million mapped reads (FPKM). The gene expression level in Jurkat T cells treated with DMSO and 0.5 μM JQ1 in the absence or presence of PHA is shown in Supplemental Table 4. The data of Jurkat cells treated with different drugs are available in the Gene Expression Omnibus (GEO) (accession code GSE205605).

### In vivo experiment

Female C57BL/6J mice (6–8 weeks old) were purchased from Beijing Vital River Laboratory Animal Technology Co. Ltd. Murine MLL-AF9 leukemia cells were from Professor Hui Cheng, and were injected into female C57BL/6J mice through the tail vein. The percentages of GFP + leukemia cells in peripheral blood were detected on 11 days after MLL-AF9 cells injection. When the proportion of GFP + cells reached 0.01–2%, mice were randomized into two groups, and intraperitoneally treated with DMSO or JQ1 (50 mg/kg) for 7 consecutive days. The percentages of PD-1 and Tim-3 on CD3 + T cells and the percentage of PD-L1 on GFP + cells in peripheral blood were detected by FACS every 2 days after DMSO or JQ1 administration. For the T-cell depletion experiment, female C57BL/6J mice were randomly grouped, and intraperitoneally treated with 200 μg IgG1 or anti-CD3 monoclonal antibody 6 days after MLL-AF9 cells were intravenously injected. Then mice treated with IgG1 or anti-CD3 monoclonal antibody were again grouped into another two groups according to the percentage of GFP + cells and were administrated with DMSO or 50 mg/kg JQ1 for 8 consecutive days.

Female NOD-Prkdcscid IL2rgtm1/Bcgen (B-NDG) mice were purchased from Biocytogen Pharmaceuticals (Beijing) Co., Ltd. 6–8-week-old female B-NDG mice were intravenously injected with 1 × 10^5^ luciferase/GFP/Nalm6 cells through the tail vein, followed by bioluminescence imaging using the IVIS imaging system after luciferin injection 4 days after Nalm6 cells administration, and the values were detected and analyzed by Living Image software. Then mice were randomized into three groups (GFP-T, DMSO-CAR19-T, and JQ1-CAR19-T) (*n* = 5) according to the bioluminescence values to ensure the equality of tumor burden among groups and were intravenously inoculated with 5 × 10^6 ^ T cells treated with DMSO or JQ1 3 days in advance. Disease progression was measured and quantified weekly by bioluminescence imaging.

All animal studies were approved by Jinan University Laboratory Animal Ethics Committee (No. 20200318-17), and were performed in compliance with institutional regulations for animal use.

### Statistical analysis

Data are presented as the mean ± standard deviation (SD). The data were analyzed by SPSS 22.0 software. All in vitro experiments consisted of three or more biological replicates per experimental group and were represented as individual data points. In vivo studies consisted of five or six mice per group. Statistical tests were performed on data from independent biological replicates. For comparisons between two groups, two-tailed unpaired Student’s *t* test or two-tailed paired Student’s *t* test was applied. For comparing three or more groups, one-way ANOVA with Bonferroni post hoc test was applied. One-way ANOVA with Dunnett post hoc test was used for comparing a control group with two or more experimental groups. For in vivo experiments, overall survival was depicted by a Kaplan–Meier curve, and the log-rank test was used to compare survival differences between the groups. *P* value < 0.05 was considered to be significant. *P < 0.05; **P < 0.01; ***P < 0.001.

## Results

### Identification of potential candidates for suppressing PD-1 expression by molecular compound screening

**Fig. 1:**
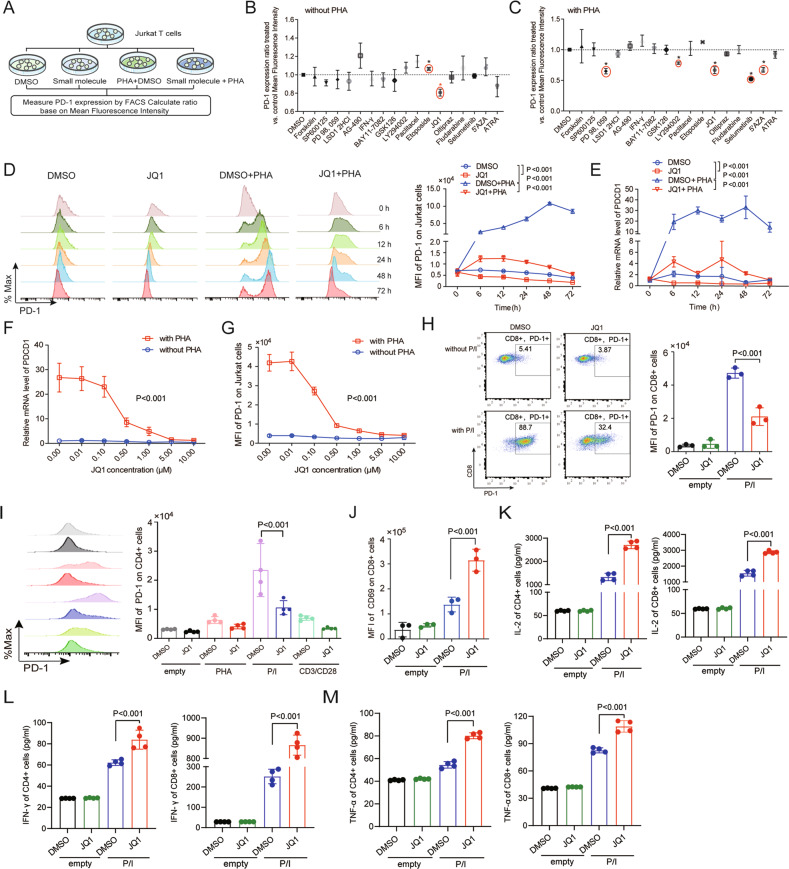
Molecular compound screening identifies JQ1 that suppresses PD-1 expression of Jurkat cell and T cell. **A** Flow diagram of the experimental design. **B** Plot of the ratio of PD-1 expression on Jurkat cells treated with the indicated compounds as detailed in Supplemental Table 5 (*n* = 3). **C** Plot of the ratio of PD-1 expression on Jurkat cells stimulated with 150 ng/mL PHA and the same compounds as that shown in Supplemental Table 5 (*n* = 3). **D** Jurkat cells were treated with 0.5 μM JQ1 with and without PHA (150 ng/mL) stimulation, and PD-1 expression was determined by FACS at the indicated time points (*n* = 4). **E** Jurkat cells were treated with 0.5 μM JQ1 with and without PHA (150 ng/mL) stimulation, and PD-1 expression was determined by qRT-PCR at the indicated time points (*n* = 3). **F** Jurkat cells were treated with indicated doses of JQ1 for 24 h in the presence or absence of PHA (150 ng/mL), PD-1 expression was determined by qRT-PCR (*n* = 5). **G** Jurkat cells were treated with indicated doses of JQ1 for 24 h in the presence or absence of PHA (150 ng/mL), PD-1 expression was determined by FACS (*n* = 4). **H** CD8 + T cells from healthy donors were treated with 0.5 μM JQ1 for 24 h with and without P/I (PMA: 20 ng/L, ionomycin: 0.5 μM) stimulation, and PD-1 expression was determined by FACS (*n* = 3). **I** CD4 + T cells from healthy donors were treated with 0.5 μM JQ1 for 24 h in the presence and absence of PHA (500 ng/ml), P/I (PMA: 20 ng/L, ionomycin: 0.5 μM), or anti-CD3/CD28 (anti-CD3: 200 ng/ml, anti-CD28: 200 ng/ml), and PD-1 expression was determined by FACS (*n* = 4). **J** CD8 + T cells from healthy donors were treated with 0.5 μM JQ1 for 24 h with and without P/I (PMA: 20 ng/L, ionomycin: 0.5 μM) stimulation, and CD69 expression was determined by FACS (*n* = 3). **K** CD4 + T cells and CD8 + T cells were treated with 0.5 μM JQ1 for 24 h, and the secretions of IL-2 were evaluated by ELISA (*n* = 4). **L** CD4 + T cells and CD8 + T cells were treated with 0.5 μM JQ1 for 24 h, and the secretions of IFN-γ were evaluated by ELISA (*n* = 4). **M** CD4 + T cells and CD8 + T cells were treated with 0.5 μM JQ1 for 24 h, and the secretions of TNF-α were evaluated by ELISA (*n* = 4). Data in (**B**–**M**) were expressed as mean ± SD. *n* = 3 or more independent biological replicates, presented as individual points. *P* value < 0.05 was considered to be significant (**D**–**G**: two-way ANOVA, **B**, **C**, **H**–**M**: one-way ANOVA with Bonferroni post hoc test).

Characterizing PD-1 regulation will help elucidate the molecular mechanisms underlying T-cell exhaustion and improve cancer treatment. Here, we conducted a molecular compound screen using the Jurkat immortalized human T-cell line to explore potential molecular candidates that inhibit PD-1 expression. A panel of 17 small-molecule compounds (Supplemental Table 5) consisting of inhibitors and activators that target different signaling pathways was used to identify potential molecular compounds that suppress PD-1 expression (Fig. 1A). To limit the potential of apoptosis induced by the small-molecule inhibitors and activators, a low concentration was chosen to treat the cells. This analysis demonstrated that only BET protein inhibitors induced PD-1 suppression, then a secondary screen using the same panel of compounds was performed in Jurkat T cells treated with phytohemagglutinin (PHA) which can induce PD-1 expression (Fig. 1B). We found that inhibitors targeting BET proteins, MEK, PI3K, and DNA methyltransferase significantly suppressed PD-1 expression (Fig. 1C). This screen revealed that JQ1 was able to suppress PD-1 expression in the presence and absence of PHA. Based on these observations, we focused on BET inhibitors as promising suppressors of PD-1 expression in T cells.

Next, we demonstrated that treatment of Jurkat T cells with JQ1 results in time- and dose-dependent suppression of PD-1 cell surface expression in the presence or absence of PHA, which correlated well with the PD-1 mRNA level (Fig. 1D–G). Furthermore, we explored the efficiency of JQ1 in suppressing PD-1 expression on Jurkat T cells treated with different concentrations of PHA. As shown in Supplemental Fig. 2A, JQ1 significantly inhibited the elevated PD-1 expression induced by PHA.

We next investigated whether BRD4 inhibition had a similar effect in primary T cells by sorting CD4 + and CD8 + T cells from healthy donors for further experiments. As expected, JQ1 substantially prevented the increase of PD-1 expression on CD8 + T cells (Fig. 1H) and CD4 + T cells treated after P/I stimulation (Fig. 1I). Furthermore, JQ1 promoted the expression of CD69, an important marker of lymphocyte activation, in CD8 + and CD4 + T cells (Fig. 1J and Supplemental Fig. 2B). To further explore whether BRD4 inhibition influences the cytokine secretion of T cells, IL-2, IFN-γ, and TNF-α levels were detected in CD4 + and CD8 + T cells. The ELISA results demonstrated that JQ1 enhanced the secretion of IL-2, IFN-γ, and TNF-α in CD4 + and CD8 + T cells after P/I stimulation (Fig. 1K–M), while significant change was only observed for IL-2 and IFN-γ by using intracellular flow cytometry (Supplemental Fig. 2C, D). In addition, JQ1 inhibited PD-1 expression in CD4 + and CD8 + T cells without inducing significant apoptosis and cell viability inhibition at 0.5 μM (Supplemental Fig. 2E, F). Collectively, these data suggest that BET inhibitor JQ1 suppresses PD-1 expression without impairing primary T-cell function.

### BRD4 inhibition suppresses PD-1 and Tim-3 expression

**Fig. 2:**
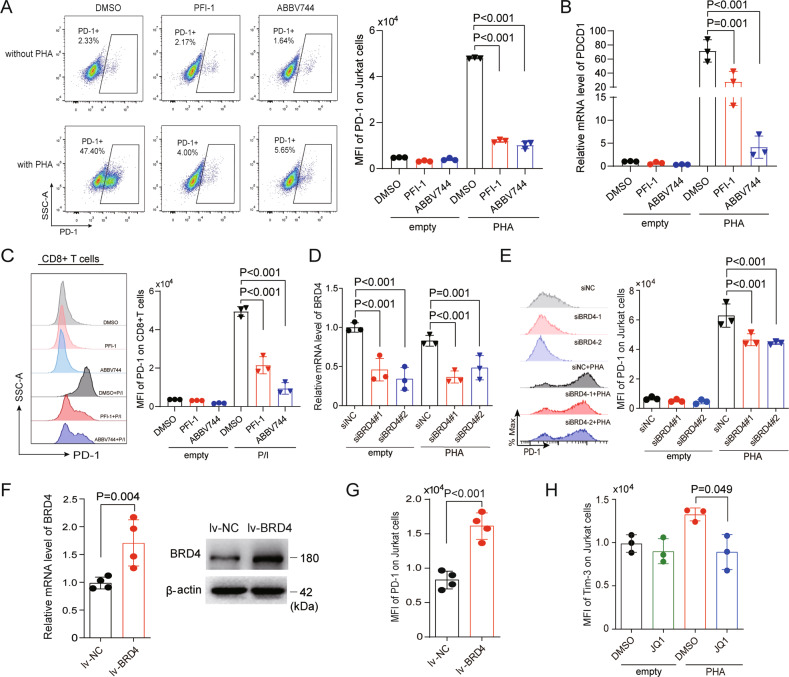
BRD4 inhibition suppresses the expression of PD-1 and Tim-3. **A** Jurkat cells stimulated with or without PHA (150 ng/mL) were treated with the BET inhibitors PFI-1 (5 μM) and ABBV-744 (2 μM) for 24 h, then PD-1 expression was determined by flow cytometry (*n* = 3). **B** Jurkat cells stimulated with or without PHA (150 ng/mL) were treated with the BET inhibitors PFI-1 (5 μM) and ABBV-744 (2 μM) for 24 h, then PD-1 expression was determined by qRT-PCR (*n* = 3). **C** Similar to Jurkat cells, CD8 + T cells were administrated with PFI-1 (5 μM), and ABBV-744 (2 μM) for 24 h stimulated with or without P/I, and PD-1 expression was determined by FACS (*n* = 3). **D** After Jurkat cells were stimulated with and without 150 ng/mL PHA after BRD4 knockdown by siRNA, BRD4 expression was examined by qRT-PCR (*n* = 3). **E** After Jurkat cells were stimulated with and without 150 ng/mL PHA after BRD4 knockdown by siRNA, PD-1 expression was determined by FACS (*n* = 3). **F** Jurkat cells were transfected by BRD4 overexpression lentivirus, then BRD4 expression was examined by qRT-PCR (*n* = 4) and western blot. **G** PD-1 expression of Jurkat cells was detected by FACS after BRD4 overexpression lentivirus was transfected (*n* = 4). **H** Jurkat cells were treated with 0.5 μM JQ1 for 24 h in the presence and absence of PHA (150 ng/mL), then the expression of Tim-3 was examined by FACS (*n* = 3). Data in (**A**–**H**) are expressed as mean ± SD. *n* = 3 or more independent biological replicates, presented as individual points. For western blot, similar results were obtained for three independent biological experiments. *P* value < 0.05 was considered to be significant (**A**–**E**, **H**: one-way ANOVA with Bonferroni post hoc test; **F**, **G**: two-tailed unpaired Student’s *t* tests).

BRD4 is one of the major bromodomain transcription factors targeted by JQ1. Similar JQ1 effects were observed in Jurkat T cells (Fig. 2A, B) and CD8 + T cells (Fig. 2C) with two other BET inhibitors, PFI-1 and ABBV-744 (a BET inhibitor which has been used in clinical trials). Thus, the suppression of PD-1 by a BET inhibitor may depend on BRD4. Indeed, the depletion of BRD4 participates in the downregulation of PD-1 expression (Fig. 2D, E and Supplemental Fig. 2G). We next sought to determine whether BRD4 overexpression increased PD-1 expression (Fig. 2F), and found that PD-1 expression was elevated by BRD4 overexpression (Fig. 2G). In addition to PD-1, JQ1 could also decrease the expression of Tim-3, another key inhibitory receptor of T-cell exhaustion (Fig. 2H). Taken together, these results further indicate that BRD4 is involved in the regulation of PD-1 and Tim-3.

### BRD4 inhibition rescues T-cell exhaustion

**Fig. 3:**
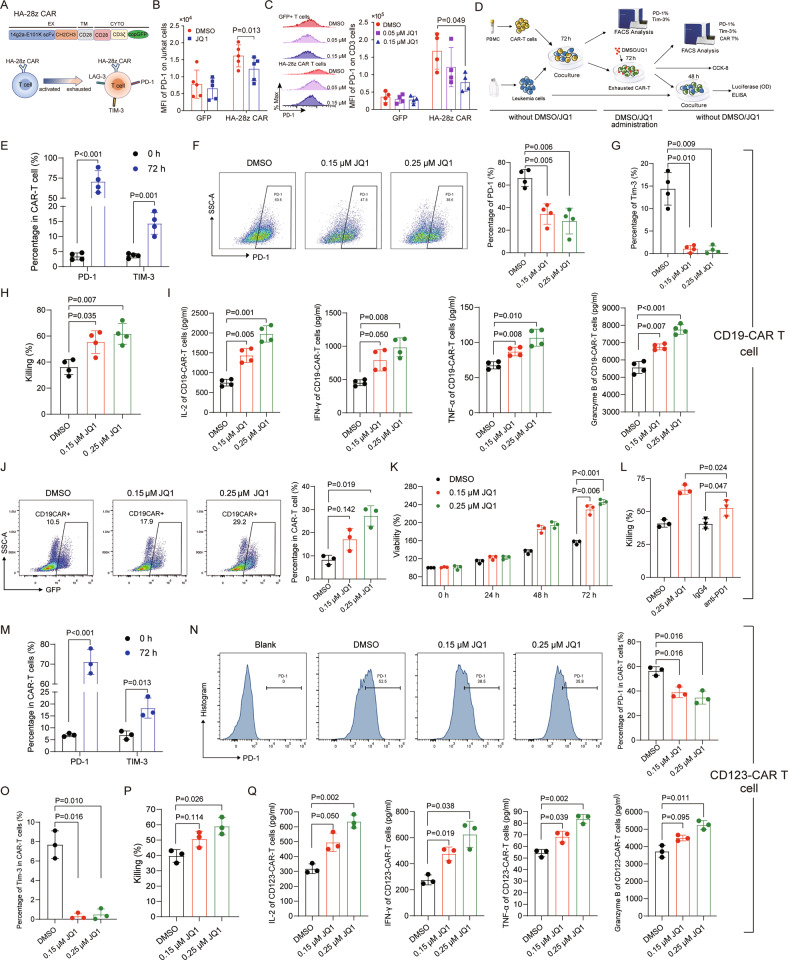
BRD4 inhibition reverses T-cell exhaustion. **A** Structural diagram of HA-28z CAR and HA-28z CAR-induced T-cell exhaustion. **B** HA-28z CAR Jurkat cells were treated with 0.5 μM JQ1 for 24 h, and PD-1 expression was determined by FACS (*n* = 5). **C** HA-28z CAR T cells positively selected by CD3 microbeads from peripheral blood mononuclear cells (PBMC) were treated with 0.05 μM and 0.15 μM JQ1 for 48 h, and PD-1 expression was determined by flow cytometry (*n* = 4). **D** Experimental design: CAR T cells were co-cultured with GFP/luciferase-expressing AML or B-ALL cells for 72 h, after eradication of leukemia cells, residual CAR T cells were collected and treated with JQ1 for another 72 h, then JQ1 was removed by centrifugation. Next, the CAR T cells in each group were counted and seeded at the same amount followed by a second co-culture with leukemia cells or CCK-8 assay. **E** The percentage of PD-1 and Tim-3 on CD19-CAR T cells after co-cultured with Nalm6-GL cells for 72 h were detected by flow cytometry (*n* = 4). **F**, **G** After being administrated with JQ1 for 72 h, the percentage of PD-1 and Tim-3 on exhausted CD19-CAR T cells were detected by flow cytometry (*n* = 4). **H** After being treated with indicated doses of JQ1 for 72 h, exhausted CD19-CAR T cells were co-cultured with Nalm6-GL cells in an E:T ratio of 1:1 for 48 h, and the remaining Nalm6-GL cells were assayed for luminescence (*n* = 4). **I** The cytokines in the supernatant of CD19-CAR T cells co-cultured with Nalm6-GL cells after JQ1 administration were detected by ELISA (*n* = 4). **J** FACS was used to monitor the percentage of CD19-CAR T cells after JQ1 administration for 72 h (*n* = 3). **K** CD19-CAR T cells were sorted, and co-cultured with Nalm6-GL for 72 h, then CD19-CAR T cells were treated with 0.15 μM or 0.25 μM JQ1, and the viability of CD19-CAR T cells was detected by CCK-8 (*n* = 3). **L** After co-cultured with Nalm6-GL cells for 72 h, CD19-CAR T cells were again co-cultured with Nalm6-GL cells in the presence of anti-PD-1 for 48 h, or were again co-cultured with Nalm6-GL cells after the treatment of 0.25 μM JQ1 for 72 h, and the remaining Nalm6-GL cells were assayed for luminescence (*n* = 3). **M** The percentage of PD-1 and Tim-3 on CD123-CAR T cells after co-cultured with MV411-GL cells for 72 h were detected by flow cytometry (*n* = 3). **N**, **O** After being administrated with JQ1 for 72 h, the percentage of PD-1 and Tim-3 on exhausted CD123-CAR T cells were detected by flow cytometry (*n* = 3). **P** After being treated with indicated doses of JQ1 for 72 h, exhausted CD123-CAR T cells were co-cultured with MV411-GL cells in an E:T ratio of 1:1 for 48 h, and the remaining MV411-GL cells were assayed for luminescence (*n* = 3). **Q** The cytokines in the supernatant of CD123-CAR T cells co-cultured with MV411-GL cells after JQ1 administration were detected by ELISA (*n* = 3). Data are expressed as mean ± SD. *n* = 3 or more independent biological replicates, presented as individual points. *P* value < 0.05 was considered to be significant (**B**–**O**: one-way ANOVA with Dunnett post hoc test; **L**: one-way ANOVA with Bonferroni post hoc test).

A recent study demonstrated that healthy T cells expressing a CAR, which incorporates the high-affinity anti-disialoganglioside (GD2) 14g2a-E101K scFv, CD3ζ, and CD28 signaling domains (HA-28z), are driven to exhaustion by the clustering of surface CAR tonic signaling in the absence of antigen (Fig. 3A) [41]. We then exploited HA-28z CAR T cells as a model to investigate the effects of JQ1 in revising T-cell exhaustion. Jurkat T cells and primary CD3 + T cells were treated with JQ1 after transduction with HA-28z CAR. We found that expression of PD-1 and TIM-3 were markedly decreased in exhausted T cells (Fig. 3B, C and Supplemental Fig. 2H).

T-cell exhaustion is characterized by significant upregulation of inhibitory receptors and loss of T-cell effector function under continuous antigen stimulation [46]. Therefore, we used the specific recognition and killing of tumor antigen by CD19-CAR T cells and CD123-CAR T cells to induce T-cells exhaustion and verify the reversal effect of JQ1 on exhausted T cells (Fig. 3D). As shown in Fig. 3E, the expression of PD-1 and TIM-3 on CD19-CAR T cells was evidently increased after co-cultured with Nalm6-GL (GFP/luciferase-expressing Nalm6) B-ALL cells, which indicated T-cell exhaustion, while JQ1 could reduce the inhibitory receptor upregulation induced by tumor antigen stimulation (Fig. 3F, G), and enhanced cytokine secretion and the killing function of exhausted T cells (Fig. 3H, I and Supplemental Fig. 2I). Obviously, we found that JQ1-treated T cells exhibited high viability, as indicated by flow cytometric analysis and CCK-8 assay (Fig. 3J, K), which was consistent with a recent study [39]. Similarly, anti-PD-1 enhanced CAR T cell efficacy in killing model of CD19-CAR T cells co-cultured with Nalm6-GL cells, but with less extent than JQ1 (Fig. 3L). We also employed a co-culture of MV411-GL (GFP/luciferase-expressing MV411) AML cells with CD123-CAR T cells which are specific targeted to AML cells. Accordingly, JQ1-treated CD123-CAR T cells exhibited a less exhausted phenotype and enhanced the killing function (Fig. 3M–Q). Taken together, these data suggested that JQ1 can reverse the exhaustion of T cells by inhibiting the expression of inhibitory receptors and restore T-cell higher viability.

### BET inhibition reverses the exhausted phenotype of T cells from AML patients

**Fig. 4:**
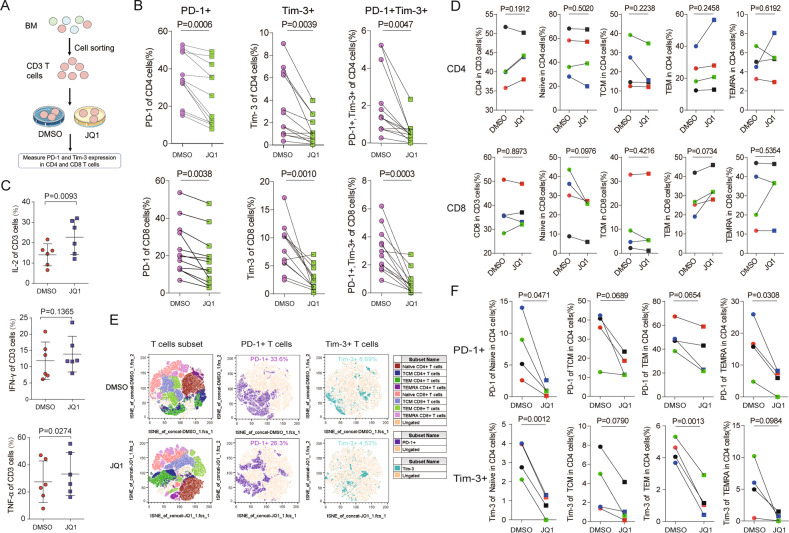
BET inhibition reverses the exhausted phenotype of T cells from AML patients. **A** Flow diagram of the experimental design. **B** CD4 + T cells (first row) and CD8 + T cells (second row) from bone marrow (BM) of newly diagnosed AML patients were treated with 0.5 μM JQ1 for 24 h, and the percentages of PD-1-positive (first column), Tim-3-positive (second column) and PD-1 and Tim-3 double-positive (third column) cells were determined by FACS. **C** CD3 + T cells from patients were treated with 0.5 μM JQ1 for 24 h, and the percentages of IL-2, IFN-γ, and TNF-α from CD3 + T cells were evaluated by intracellular flow cytometry. **D** CD3 + T cells from AML patients (*n* = 4 samples) were treated with 0.5 μM JQ1 for 24 h, and the percentages of the CD4 + (upper), and CD8 + (lower) subpopulations were shown with differences in the phenotypic distribution of naive (CCR7 + CD45RA + , second column), TCM (CCR7 + CD45RA − , third column), TEM (CCR7 − CD45RA − , fourth column), and TEMRA (CCR7 − CD45RA + , right) T-cell subsets. **E** © Concatenated files representing AML samples in the DMSO group (upper) and JQ1 (lower) groups projected in tSEN plots after gating on lymphocyte cells. Distribution of naive, TCM, TEM, and TEMRA CD4 + T cells were shown as dark red, dark yellow, dark green, and dark purple, respectively, and the distribution of naive, TCM, TEM, and TEMRA CD8 + T cells were shown as light red, light yellow, light green, and light purple, respectively. **F** CD3 + T cells from AML patients (*n* = 4 samples) were treated with 0.5 μM JQ1 for 24 h, and the percentages of PD-1 positive (upper row) and Tim-3 positive (lower row) cells in naive (left), TCM (second column), TEM (third column), and TEMRA (right) CD4 + T cells were determined by FACS. Data are expressed as mean ± SD. *P* value < 0.05 was considered to be significant (two-tailed paired Student’s *t* tests).

Previous studies have demonstrated T-cell dysfunction in patients with hematologic malignancies [8, 47], and blockade of PD-1 and Tim-3 was able to reverse T-cell exhaustion and restore anticancer immunity [48]. Therefore, we investigated the effects of JQ1 in T cells from AML patients. CD3 + T cells from patients with newly diagnosed AML were sorted and treated with JQ1 or vehicle control (DMSO) for 24 hours, then PD-1 and Tim-3 levels were measured at the end of drug treatment by FCM (Fig. 4A and Supplemental Fig. 3A). As shown in Fig. 4B, a marked reduction of PD-1 and Tim-3 on CD4 + and CD8 + T cells treated with JQ1 compared with vehicle control was observed. In addition, double-positive PD-1+Tim-3 + T cells were significantly decreased by JQ1 treatment. Collectively, these data suggest that suppression of PD-1 and Tim-3 expression by JQ1 may reverse T-cell exhaustion in AML.

We hypothesized that JQ1 could restore the function of exhausted T cells. To verify this hypothesis, we first evaluated the IL-2, IFN-γ, and TNF-α levels in CD3 + T cells from AML patients after JQ1 treatment. JQ1-treated CD3 + T cells from AML patients demonstrated increased cytokine secretion although not all of the tested cytokine show statistically significant (Fig. 4C and Supplemental Fig. 3B). Next, we explored the effects of JQ1 on T-cell differentiation status. The expression of PD-1 and Tim-3 in naive, central memory (TCM), effector memory (TEM), and the terminal differentiated effector memory (TEMRA) subset of CD4 + and CD8 + T cells from AML patients was analyzed (Supplemental Fig. 3C). We observed that JQ1 treatment did not increased the frequency of the TCM, TEM, and TEMRA subsets in the CD4 + and CD8 + T-cell populations in AML patients (Fig. 4D). Instead, we found that the level of PD-1 and Tim-3 in the CD4 + and CD8 + T-cell subsets was different between the two culture conditions. In particular, the PD-1 and Tim-3 levels were substantially lower in the JQ1-treated TEM subset (Fig. 4E, F and Supplemental Fig. 3D). These data further support our hypothesis that BET bromodomain inhibition partially mitigates T-cell exhaustion in AML.

### BRD4 inhibition rescues T-cell exhaustion via the NFAT2 signaling pathway

**Fig. 5:**
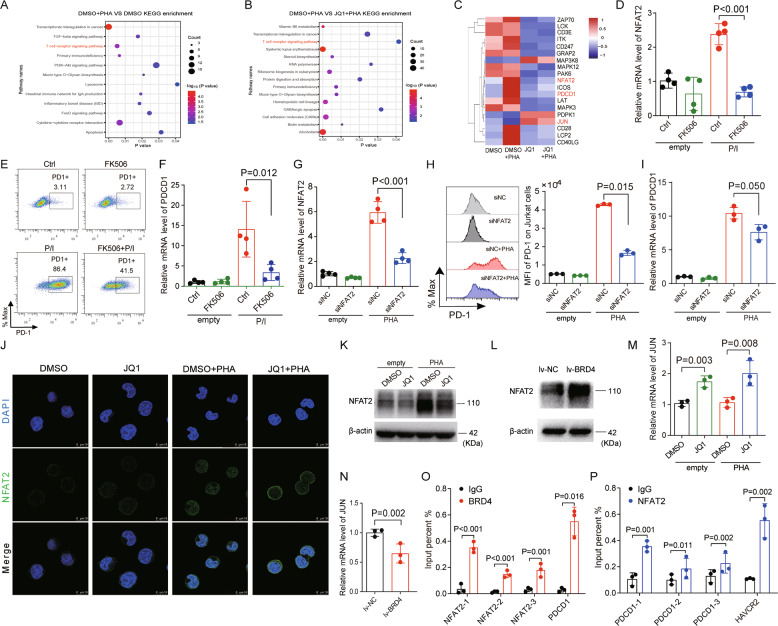
BRD4 inhibition suppresses the expression of PD-1 and Tim-3 via NFAT2. **A** The top 12 enriched terms of the Kyoto Encyclopedia of Genes and Genomes (KEGG) enrichment pathway analyses were shown in the bubble plot (DMSO + PHA VS DMSO). The KEGG pathways was identified by R package “clusterProfiler”, and *P* < 0.05 was considered statistically significant. **B** Scatter plot of top 15 enriched KEGG pathways were shown in the bubble plot (DMSO + PHA VS JQ1 + PHA). **C** A heatmap of the expression of genes involved in the TCR signaling pathway in Jurkat cells was analyzed by RNA-seq. **D** CD8 + T cells were cultured with 100 ng/ml FK506 for 24 h in the presence and absence of P/I (PMA: 20 ng/L, ionomycin: 0.5 μM), NFAT2 expression was determined by qRT-PCR (*n* = 4). **E** PD-1 expression of CD8 + T cells treated with 100 ng/ml FK506 for 24 h in the presence and absence of P/I was determined by FACS (*n* = 4). **F** PD-1 expression of CD8 + T cells treated with 100 ng/ml FK506 for 24 h in the presence and absence of P/I was determined by qRT-PCR (*n* = 4). **G** Jurkat cells were stimulated with and without PHA (150 ng/mL) after NFAT2 knockdown, then qRT-PCR was used to examine the expression of NFAT2 (*n* = 4). **H** PD-1 expression of Jurkat cells stimulated with and without PHA (150 ng/mL) after NFAT2 knockdown was determined by FACS (*n* = 3). **I** PD-1 expression of Jurkat cells stimulated with and without PHA (150 ng/mL) after NFAT2 knockdown was determined by qRT-PCR (*n* = 3). **J** Jurkat cells were treated with 0.5 μM JQ1 for 24 h in the presence and absence of PHA (150 ng/mL), and NFAT2 expression was detected by immunofluorescence. **K** Jurkat cells were treated with 0.5 μM JQ1 for 24 h in the presence and absence of PHA (150 ng/mL), and NFAT2 expression was detected by western blot. **L** NFAT2 expression was examined by western blot after overexpressing BRD4 in Jurkat cells. Shown are the representative images of three separate experiments. **M** Jurkat cells were treated with 0.5 μM JQ1 for 24 h in the presence and absence of PHA (150 ng/mL), the expression of JUN was examined by qRT-PCR (*n* = 3). **N** JUN expression was detected by qRT-PCR after BRD4 overexpression in Jurkat cells (*n* = 3). **O** Chromatin samples from Jurkat cells stimulated with 150 ng/ml PHA for 24 h were immunoprecipitated with anti-BRD4 antibody, and enrichment of BRD4 at the promoter regions of NFAT2 and PDCD1 was measured by qRT-PCR (*n* = 3). **P** The chromatin samples were stimulated with 150 ng/ml PHA for 24 h and immunoprecipitated with an anti-NFAT2 antibody, enrichment of NFAT2 at the promoter regions of PDCD1 and HAVCR2 was measured by qRT-PCR (*n* = 3). Data are expressed as mean ± SD. *n* = 3 or more independent biological replicates, presented as individual points. *P* value < 0.05 was considered to be significant (**D**, **F**–**I**, **M**, **O**, **P**: one-way ANOVA with Bonferroni post hoc test; **N**: two-tailed unpaired Student’s *t* tests).

To gain mechanistic insight into the regulation of PD-1 by BET bromodomain inhibition, we performed RNA sequencing (RNA-seq) in DMSO or JQ1-treated Jurkat T cells. Kyoto Encyclopedia of Genes and Genomes (KEGG) pathway analysis of differentially expressed genes identified T-cell receptor (TCR) signaling as the candidate pathway downstream of BET inhibition (Fig. 5A, B and Supplemental Fig. 4A–C). Among the genes dysregulated in the TCR signaling pathway upon JQ1 treatment, we chose NFAT2 as a target gene because NFAT2 has been reported to control T-cell exhaustion by regulating the expressions of key inhibitory receptors that dampen TCR signaling. We further found that the expression of NFAT2 significantly declined in JQ1-treated Jurkat T cells (Fig. 5C).

Based on these results, we next inhibited NFAT2 expression in CD8 + T cells with the NFAT2 inhibitor FK506 (Fig. 5D). As expected, the expression of PD-1 was inhibited by FK506 treatment in primary T cells (Fig. 5E, F). Similar results were observed in Jurkat T cells (Supplemental Fig. 4D, E), and the expression of Tim-3 was also suppressed (Supplemental Fig. 4F). We also observed superiority of combining JQ1 and FK506 over the single agents on PD-1 expression (Supplemental Fig. 4G). We then assessed the expression of PD-1 upon NFAT2 deletion in Jurkat T cells by RNA interference, and confirmed that PD-1 expression was significantly decreased in siRNA-NFAT2 treated cells compared with negative control cells (Fig. 5G–I), which was consistent with the results from JQ1 treatment. Moreover, we found that JQ1 reduced NFAT2 expression in Jurkat T cells using immunofluorescence, western blot, and qRT-PCR analysis (Fig. 5J, K and Supplemental Fig. 4H, I). Similar effects were obtained with another BET inhibitor, PFI-1 (Supplemental Fig. 4J), and an opposite trend was observed after BRD4 overexpression (Fig. 5L). Collectively, these data indicated that BRD4 inhibition may inhibit key inhibitory receptors by suppressing NFAT2 expression.

In addition, JUN was increased by BRD4 inhibition (Fig. 5M), which has been reported to rescue exhaustion in HA-28z CAR T cells [41]. In contrast, overexpression of BRD4 downregulated JUN expression, and promoted NFAT2 and PD-1 expression (Figs. 2G and 5N, L), indicating that NFAT2 and JUN were downstream targets of BRD4. In addition to PD-1, RNA-seq data found that JQ1 also decreased the expression of Tim-3 in Jurkat T cells (Supplemental Fig. 4K), which was further confirmed by FACS analysis (Fig. 2H), suggesting that BRD4/NFAT2 may also involve in the induction of Tim-3 expression. We then assessed whether NFAT2 and PD-1 were direct targets of BRD4 by chromatin immunoprecipitation (ChIP) analysis in Jurkat T cells, and found that BRD4 bond to the NFAT2 and PDCD1 (encoding PD-1) promoters (Fig. 5O). Moreover, NFAT2 ChIP assays showed a significant association between NFAT2 and the promoters of PDCD1 and HAVCR2 (encoding Tim-3) (Fig. 5P). These data suggested that BRD4 and NFAT2 may be involved in JQ1-mediated suppression of PD-1 and Tim-3.

### BET inhibitor suppresses the PD-L1/PD-1 pathway and enhances the elimination of AML in vitro and in vivo

**Fig. 6:**
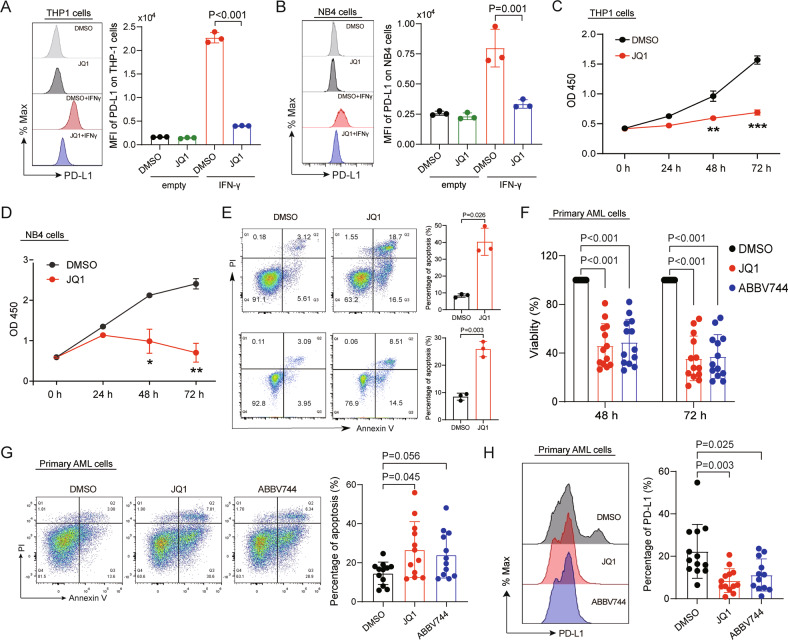
BET inhibitor suppresses PD-L1 expression and cell growth and promotes cell apoptosis in AML. **A** THP1 cells were treated with 0.5 μM JQ1 for 24 h in the absence and presence of 20 ng/ml IFN-γ, PD-L1 expression was determined by FACS (*n* = 3). **B** NB4 cells were treated with 0.5 μM JQ1 for 24 h in the absence and presence of 20 ng/ml IFN-γ, PD-L1 expression was determined by FACS (*n* = 3). **C** THP1 cells were treated with 0.5 μM JQ1 for 72 h, the proliferation assay was evaluated by CCK-8 assay at the indicated time point (*n* = 3). **D** NB4 cells were treated with 0.5 μM JQ1 for 72 h, the proliferation assay was evaluated by CCK-8 assay at the indicated time point (*n* = 3). **E** THP1 cells (upper) and NB4 cells (lower) were treated with 0.5 μM JQ1 for 24 h, and apoptosis was determined by FACS (*n* = 3). **F** The CD3− cells isolated from the bone marrow of newly diagnosed AML patients using human CD3 microbeads were administrated with 0.5 μM JQ1 and 2 μM ABBV-744 for 48 h, then the proliferation was assayed by CCK-8 experiment at the indicated time points (*n* = 13 samples). **G** The apoptosis of CD3− cells isolated from the bone marrow of newly diagnosed AML patients was detected by FACS (*n* = 12 samples). **H** PD-L1 expression of CD3− cells isolated from the bone marrow of newly diagnosed AML patients were detected by FACS (*n* = 13 samples). Data were expressed as mean ± SD. *n* = 3 or more independent biological replicates, presented as individual points. *P* value < 0.05 was considered to be significant (**A–D**, **F**–**H**: one-way ANOVA with Bonferroni post hoc test; **E**: two-tailed unpaired Student’s *t* tests).

Previous studies had proved that BET inhibitor could promote anticancer immunity by suppressing PD-L1 expression in solid tumor or lymphoma [49, 50], but have not yet been reported in AML. Therefore, we analyzed the effects of JQ1 and ABBV-744 on AML cell lines and primary AML cells. We observed that JQ1 sharply diminished the IFN-γ-induced upregulation of PD-L1 in THP1, NB4 cells and HEL cells (Fig. 6A, B and Supplemental Fig. 5A). Moreover, the proliferation of THP1 cells, NB4 cells, and HEL cells were inhibited significantly by JQ1, especially after 72 hours treatment (Fig. 6C, D and Supplemental Fig. 5B). Further studies revealed that JQ1 increased apoptosis in THP1 cells, NB4 cells and HEL cells (Fig. 6E and Supplemental Fig. 5C), which was consistent with previous studies [51, 52]. Similar results were shown in CD3- cells from newly diagnosed AML patients treated with JQ1 or ABBV-744 (Fig. 6F–H). Taken together, these results suggested that in addition to suppress growth of cancer cells, BET inhibitors may also enhance the immunity of AML patients by suppressing the expression of PD-L1 on AML cells and the expression of PD-1 and Tim-3 on exhausted T cells.

**Fig. 7:**
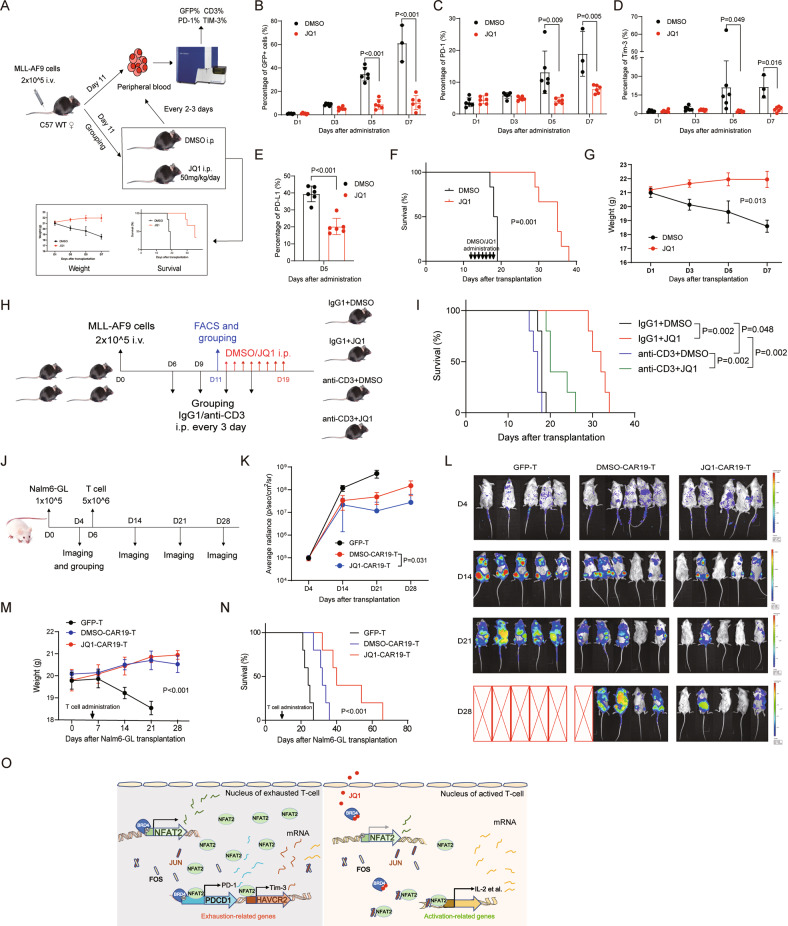
BET inhibitor suppresses PD-1 and Tim-3 expression, and prolongs survival in murine AML and B-ALL leukemia model. **A** Structural diagram of in vivo experiment treated with JQ1 (*n* = 6 mice per group in this experiment, *n* = 3 mice per group in the repeated experiment, *n* (total) = 9 mice). **B** The percentages of GFP + leukemia cells in peripheral blood were detected by FACS every 2 days after DMSO or JQ1 administration (*n* = 6 mice per group). **C** The expression of PD-1 on CD3 + T cells in peripheral blood was detected by FACS every 2 days after DMSO or JQ1 administration (*n* = 6 mice per group). **D** The expression of Tim-3 on CD3 + T cells in peripheral blood was detected by FACS every 2 days after DMSO or JQ1 administration (*n* = 6 mice per group). **E** The expression of PD-L1 on GFP + T cells in peripheral blood was detected by FACS every 2 days after DMSO or JQ1 administration (*n* = 6 mice per group). **F** Kaplan–Meier curve after MLL-AF9 leukemia cells transplantation (*n* = 6 mice per group, statistical significance calculated using a log-rank test). **G** Weight change of each mouse in DMSO or JQ1-treated group (*n* = 6 mice per group). **H** Structural diagram of in vivo experiment treated with anti-CD3 and JQ1 (*n* (total) = 5 mice per group). **I** Kaplan–Meier curve after MLL-AF9 leukemia cells transplantation (*n* = 5 mice per group). **J** Treatment schedule and experimental design. B-NDG mice were injected with 1 × l0^5^ Nalm6 cells on day 0, followed by 5 × l0^6^ GFP-T or DMSO-CAR19-T or JQ1-CAR19-T on day 6. Bioluminescence imaging was performed weekly after T-cell administration (*n* = 5 mice per group in this experiment, *n* = 6 mice per group in the repeated experiment, *n* (total) = 11 mice). **K** The dorsal BLI signal is displayed for individual mice in each treatment group (*n* = 5 mice per group). **L** D4-28 bioluminescence imaging of tumor growth (*n* = 5 mice per group). **M** Weight change of each mouse in each treatment group (*n* = 5 mice per group). **N** Kaplan–Meier survival plot for mice receiving GFP-T or CAR T cells pretreated with DMSO or JQ1 (*n* = 5 mice per group). **O** Schematic representation of the mechanism underlying BRD4 inhibition leading to improve T-cell efficacy. Left: BRD4 and NFAT2 involve in the transcription of PDCD1 and HAVCR2. Right: BRD4 inhibitor JQ1 blocks the binding of BRD4 to NFAT2 promoter to prevent the transcription of NFAT2, which inhibits the transcription of PDCD1 and HAVCR2. Besides, JQ1 blocks the binding of BRD4 to the PDCD1 promoter, thus directly inhibiting the expression of PD-1. In short, BRD4 inhibitor inhibits the expression of PD-1 and Tim-3, and increases the secretion of cytokines may partly through NFAT2 signaling pathway. Data are expressed as mean ± SD. *n* = 3 or more independent biological replicates, presented as individual points. *P* value < 0.05 was considered to be significant (**B**–**D**: one-way ANOVA with Bonferroni post hoc test; **G**, **K**, **M**: two-way ANOVA; **E**: two-tailed unpaired Student’s *t* tests; **F**, **I**, **N**: log-rank teat).

There have been reported that JQ1 effective against AML cells and lengthen lifespan of AML mice [53, 54]. However, whether the effects JQ1 involve the reversal of T-cell exhaustion in AML has not been addressed. We then used a murine AML model driven by MLL-AF9 to determine the effects of JQ1 treatment on anti-leukemia immunity in vivo (Fig. 7A and Supplemental Fig. 6A). JQ1 treatment also reduced the leukemia burden (GFP + leukemic cells) in peripheral blood (Fig. 7B). In addition, JQ1 treatment significantly reduced the expression of PD-1 and Tim-3 on mice CD3 + T cells (Fig. 7C, D), while we did not see a clear upregulation of the overall T cells in mice’s peripheral blood. Remarkably, the survival rate was significantly higher in JQ1-treated group than in the control mice (Fig. 7E–G). We asked whether T cell is required for the effects of JQ1, to this end, we performed T-cell depletion in MLL-AF9 AML mouse model. Analysis of mice overall survival revealed that T-cell depletion by anti-CD3 treatment blocked the therapeutic efficacy of JQ1 (Fig. 7H, I and Supplemental Fig. 6B).

Moreover, we used a luciferase/GFP/Nalm6-breaing mouse leukemia model to test the reverse efficacy of BET inhibitor on T-cell exhaustion (Fig. 7J), and found that in all treated mice, CAR T cells pretreated by JQ1 produced a significantly lowed tumor burden and longer remission, as well as prolonged survival, compared with the control group (Fig. 7K–N). Taken together, these data showed the anti-leukemia effects of BET inhibitor by dual action on exhausted T cells and AML cells.

## Discussion

In this study, to identify targeted inhibitors that can overcome the T-cell exhaustion mediated by PD-1, we first performed a screen of small-molecule inhibitors to identify potential factors that are involved in PD-1 regulation and T-cell exhaustion. We found that JQ1 is a critical inhibitor that suppresses PD-1 expression in T cells. JQ1 is a BET inhibitor that has been reported to exert anticancer activity by targeting c-Myc and PD-L1 in cancer cells [55, 56]. In this study, we reported that targeting BET proteins can compromise PD-1-mediated suppression of T cells, and found that BET inhibitors can suppress the expression of PD-L1 in AML cells. Thus, BET inhibitors may regulate anti-leukemia immunity by suppressing the expression of both PD-L1 in cancer cells and PD-1 in T cells. Given the fact that BET inhibitors have clear anticancer activity in a variety of solid tumors and hematologic malignancies [57–59], our findings provide evidence and a rationale for the development of novel therapeutic strategies to improve immune responses in AML based on targeting BRD4.

PD-1 blockade can reverse T-cell exhaustion and has prominent therapeutic benefits in an expanding list of cancers, and co-inhibiting Tim-3 enhances the anticancer effects of PD-1 blockade [60]. Thus, PD-1 blockade may induce incomplete rescue of T-cell exhaustion. We found that inhibition of BRD4 with chemical inhibitors markedly reduced PD-1 and Tim-3 expression on Jurkat T cells, primary T cells, HA-28z CAR T cells, and T cells from patients with AML, and siBRD4 could suppress PD-1 expression on Jurkat T cells. These findings highlighted that BRD4 may be directly involved in the induction of PD-1 expression. However, a role for other BET family proteins in PD-1 expression cannot be excluded.

T cells express a high level of PD-1 in both the activation and exhaustion state. However, PD-1 primarily affects functions of repetitive antigen exposure to effector T cells but does not induce an exhaustion program at the early stage of T-cell activation [61]. In this study, we showed that JQ1 decreased PD-1 in both T-cell activation and exhaustion cell models, but it did not repress T-cell activity, as evidenced by CD69 expression, cell viability, and cytokines secretion. This finding may indicate that BET bromodomain inhibition compromises excessive PD-1-mediated suppression of T cells rather than repressing T-cell activation. Using a tonically signaling CAR that can induce the hallmark characteristics of exhaustion, we found that JQ1-treated cells exhibited low PD-1 expression. In line with this result, JQ1 dramatically impaired the expression of PD-1 and Tim-3 and enhanced cytokine production in T cells from patients with AML. Through our investigation of JQ1 effects in CD19-CAR T-cell exhaustion model, we found that BET inhibitor-treated CAR T cells have enhanced anti-leukemia potency and higher viability. Importantly, JQ1 increased the cytotoxicity of CD123-CAR T cells (specifically targeted to AML cells) under the same condition. Furthermore, we have also obtained additional data demonstrating that anti-PD-1 enhances CD19-CAR T-cell efficacy in the CAR T-cell exhaustion model (Fig. 3). Although PD-1 blockade by antibody enhanced anti-leukemia function of CD19-CAR T cell, this effect was moderate and with less extent than JQ1. This result is in agreement with the effect of JQ1 and also suggests the involvement of other inhibitory receptors (e.g., Tim-3) in JQ1-mediated amelioration of CAR T-cell exhaustion. These findings suggest the function of BET inhibitors to prevent T-cell exhaustion partially by negatively regulating PD-1 expression. Nevertheless, further research is needed to determine whether BET inhibitors are capable of fully reprograming exhausted T cells.

The effects of JQ1, at least in part, result from transcriptional modulation of NFAT (Fig. 5). Here, our data demonstrated that the TCR signaling pathway was also involved in these effects. Downstream targets of TCR signaling, such as NFAT and AP-1, are responsible for the induction of PD-1 transcription during cancer progression. A study has also reported that T-cell exhaustion can be induced from partner-less NFAT in the absence of AP-1 [62]. Therefore, BRD4 may upregulate PD-1 expression through NFAT2. Indeed, ChIP assays showed that BRD4 can bind to the NFAT2 and PDCD1 promoters, and NFAT2 can bind to the PDCD1 and HAVCR2 promoters. In addition, it has recently been shown that BRD4 binds to the super-enhancer region near HAVCR2 encoding Tim-3 [34]. These results also indicate that BRD4 may be directly involved in regulating PD-1 and Tim-3 expression. Moreover, CAR T cells engineered to overexpress the canonical AP-1 factor JUN have demonstrated enhanced anticancer potency and the ability to resist exhaustion [41]. Consistent with this finding, our study indicates that JQ1 treatment markedly increases JUN protein expression. However, the relationship between an elevated JUN level and PD-1 suppression induced by BRD4 inhibition requires further investigation. Thus, we revealed that BRD4 inhibition exerts an anti-leukemia effect through the PD-1-regulatory pathway in AML (Fig. 7O).

Although monoclonal antibody (mAb) blockade of PD-1/PD-L1 has demonstrated significant clinical benefit for various types of human cancers, antibody-based immunotherapies have several limitations such as the high production cost of antibodies, immune-related adverse effects, and drug resistance [63, 64]. Therefore, small-molecule inhibitors may provide a promising alternative or complementary therapeutic for mAbs to block the PD-1/PD-L1 pathway. BET inhibitors have been demonstrated to possess anticancer activities in a subset of patients with hematologic malignancies in clinical trials [37, 58]. Recent data have also supported the idea that BRD4 is directly involved in the regulation of T-cell activity [39, 40]. Inhibition of BRD4 has also been shown to restore anticancer immunity by reducing PD-L1 expression on cancer cells [10]. In addition, JQ1 was shown to enhance the persistence and anticancer effects of T cells by suppressing BATF [40]. In this study, our findings raise the possibility of targeting the PD-1 regulatory pathway using a BET inhibition. BRD4 inhibition prevented PD-1-mediated T-cell exhaustion as evidenced by repressed PD-1 expression and increased IL-2 production in primary T cells. Although there are studies that show the importance of PD-1 in T-cell exhaustion, there is also evidence suggesting that PD-1 might not be the dominant driver of T-cell exhaustion [25, 65]. Apart from inhibition of PD-1 expression, Tim-3 may also play a crucial role in regulating genes involved in T-cell exhaustion. Indeed, the lower Tim-3 level in JQ1-treated exhausted T cells would further improve the T-cell functional capacity, as co-inhibiting Tim-3 has been shown to enhance the anticancer effects of PD-1 blockade [22]. Given the suppressed effects of JQ1 in PD-1 expression as well as Tim-3 expression, it is possible that targeting upstream regulators BRD4/NFAT other than PD-1 may have a better response in rescuing exhausted T cells. A more detailed understanding of the molecular mechanisms involving BRD4 and NFAT in T-cell exhaustion may provide opportunities to better understand the effects of BET inhibition in cancer immunotherapy.

In conclusion, we showed that BET bromodomain inhibition diminished the expression of the inhibitory receptors PD-1 and Tim-3, which correlated with T-cell exhaustion. We demonstrated that BET inhibitor treatment decreased BRD4 and NFAT2 expression, as well as reversed the exhaustion of T cells. Moreover, we reported that BET inhibitors suppressed PD-L1 expression and cell growth in AML cells. Besides providing novel insights into the molecular mechanisms of BRD4 inhibition in the regulation of PD-1 expression and PD-1-mediated inhibition of T-cell function, this study inspires thought on the dual effect of target inhibition BRD4 for cancer immunotherapy.

## Supplementary information

SUPPLEMENTAL MATERIAL

Supplemental Table 4

Full length western blots

aj-checklist

## Data Availability

The data that support the findings of this study are available from the corresponding author upon reasonable request.
