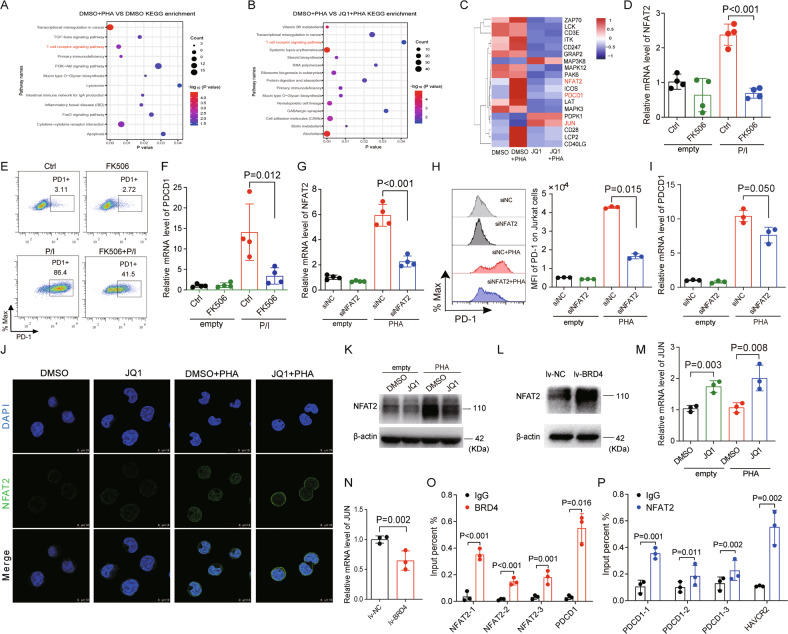# Correction to: BET bromodomain inhibition rescues PD-1-mediated T-cell exhaustion in acute myeloid leukemia

**DOI:** 10.1038/s41419-022-05204-x

**Published:** 2022-08-29

**Authors:** Mengjun Zhong, Rili Gao, Ruocong Zhao, Youxue Huang, Cunte Chen, Kehan Li, Xibao Yu, Dingrui Nie, Zheng Chen, Xin Liu, Zhuandi Liu, Shaohua Chen, Yuhong Lu, Zhi Yu, Liang Wang, Peng Li, Chengwu Zeng, Yangqiu Li

**Affiliations:** 1 Key Laboratory for Regenerative Medicine of Ministry of Education, Institute of Hematology, Jinan University, 510632 Guangzhou, P. R. China; 2 Center for Cell Regeneration and Biotherapy, Guangzhou Institutes of Biomedicine and Health, Chinese Academy of Sciences, 510530 Guangzhou, P. R. China; 3 Department of Hematology, First Affiliated Hospital, Jinan University, 510632 Guangzhou, P. R. China; 4 Department of Oncology, First Affiliated Hospital, Jinan University, 510632 Guangzhou, P. R. China

**Keywords:** Acute myeloid leukaemia, Preclinical research

Correction to: *Cell Death and Disease* 10.1038/s41419-022-05123-x, published online 02 August 2022

The original version of this article unfortunately contained some mistakes. After the publication of this paper in Cell Death & Disease in 2022, the authors noticed errors after re-reviewing this manuscript. The authors found that an incorrect image for the β-actin of Figure 5L was inadvertently included, which was different from the image of β-actin in the full- length western blots. As shown in the original Figure 5L, the 5 seconds-exposure image of β-actin was uploaded, while the image of β-actin exposure for 7 seconds was uploaded in the full-length western blots. And the molecular weight of NFAT2 (180 kDa should be 110 kDa) in Figure 5L was marked by mistake. Also, the gene “ICOS” has been mistakenly shown twice in Figure 5C, and the gene “ICOS” below “JUN” should be corrected to “CD28”. The authors confirm that the original RNA-seq data is correct. The correct figure (Figure 5) is shown below. In the “Flow cytometric analysis” of the “Materials and Methods” section (line 3 in the “Flow cytometric analysis” section on page 2), the product number of the antibody PE mouse anti-human CD279 was mismarked as “561272”, and should be corrected to “560795”. The correction does not affect the conclusions of the above paper. The authors apologize for the mistakes and any inconvenience caused.

The original article has been corrected.