# Supplementary material for: BET bromodomain inhibition rescues PD-1-mediated T-cell exhaustion in acute myeloid leukemia
Source: Cell Death Dis. 2022 Aug 2;13(8):671. doi: 10.1038/s41419-022-05123-x (PMC9346138; doi:10.1038/s41419-022-05123-x)
Supplement: Supplementary file 1 — SUPPLEMENTAL MATERIAL [file 41419_2022_5123_MOESM1_ESM.doc]

**Supplemental Table 1. Clinical information relevant to AML**

| **ID** | **Sex** | **Age** | **Used in Fig.** |
| --- | --- | --- | --- |
| 1 | female | 72 | Fig. 4B |
| 2 | male | 69 | Fig. 4B |
| 3 | male | 72 | Fig. 4B |
| 4 | female | 88 | Fig. 4B |
| 5 | male | 72 | Fig. 4B, Fig. 4D, Fig. 4E, Fig. 4F, Fig. S2C |
| 6 | female | 47 | Fig. 4B |
| 7 | female | 43 | Fig. 4B |
| 8 | female | 36 | Fig. 4B |
| 9 | male | 24 | Fig. 4B |
| 10 | male | 76 | Fig. 4B, Fig. 4D, Fig. 4E, Fig. 4F, Fig. S2C |
| 11 | male | 54 | Fig. 4B, Fig. 4D, Fig. 4E, Fig. 4F, Fig. S2C |
| 12 | male | 33 | Fig. 4B, Fig. 4D, Fig. 4E, Fig. 4F, Fig. S2C |
| 13 | male | 21 | Fig. 6F, Fig. 6H |
| 14 | female | 65 | Fig. 6F, Fig. 6H |
| 15 | male | 58 | Fig. 6F, Fig. 6G, Fig. 6H |
| 16 | male | 72 | Fig. 6F, Fig. 6G, Fig. 6H |
| 17 | male | 85 | Fig. 6F, Fig. 6G, Fig. 6H |
| 18 | male | 79 | Fig. 6F, Fig. 6G, Fig. 6H |
| 19 | female | 67 | Fig. 6F, Fig. 6G, Fig. 6H |
| 20 | female | 43 | Fig. 6F, Fig. 6G, Fig. 6H |
| 21 | female | 26 | Fig. 6F, Fig. 6G, Fig. 6H |
| 22 | female | 35 | Fig. 6F, Fig. 6G, Fig. 6H |
| 23 | male | 33 | Fig. 6F, Fig. 6G, Fig. 6H |
| 24 | male | 62 | Fig. 6F, Fig. 6G, Fig. 6H |
| 25 | male | 38 | Fig. 6F, Fig. 6G, Fig. 6H |
| 26 | male | 51 | Fig. 6G |
| 27 | male | 68 | Fig. S4B |
| 28 | female | 56 | Fig. S4B |
| 29 | male | 59 | Fig. S4B |

**Supplemental Table 2. Primer sequences used in this study**

1. **RT-qPCR primers**

| **Genes** | **Forward primer (5’-3’)** | **Reverse primer (5’-3’)** |
| --- | --- | --- |
| β-actin | TTGTTACAGGAAGTCCCTTGCC | ATGCTATCACCTCCCCTGTGTG |
| PD-1 | CGTGGCCTATCCACTCCTCA | ATCCCTTGTCCCAGCCACTC |
| NFAT2 | AGATGGAAGCGAAAACTGAC | CAGGGATCAAGGAAAATGCA |
| BRD4 | AGGCAAAAGGAAGAGGACG | CGATGCTTGAGTTGTGTTTGG |
| c-Jun | CTGCAAAGATGGAAACGACCT | GGATTATCAGGCGCTCCAG |

1. **ChIP-qPCR primers**

| **Genes** | **Forward primer (5’-3’)** | **Reverse primer (5’-3’)** |
| --- | --- | --- |
| β-actin | TTGTTACAGGAAGTCCCTTGCC | ATGCTATCACCTCCCCTGTGTG |
| PD-1 | CGTGGCCTATCCACTCCTCA | ATCCCTTGTCCCAGCCACTC |
| NFAT2 | AGATGGAAGCGAAAACTGAC | CAGGGATCAAGGAAAATGCA |
| BRD4 | AGGCAAAAGGAAGAGGACG | CGATGCTTGAGTTGTGTTTGG |
| c-Jun | CTGCAAAGATGGAAACGACCT | GGATTATCAGGCGCTCCAG |

**Supplemental Table 3. siRNA sequences used in this study**

| **Target Genes** | **Sequences (5’-3’)** |
| --- | --- |
| NFAT2#1 | GGUCAUUUUCGUGGAGAAATT |
| NFAT2#2 | GAAACUCCGACAUUGAACUTT |
| BRD4#1 | AGATTGAAATCGACTTTGA |
| BRD4#2 | TGAGCACAATCAAGTCTAA |

**Supplemental Table 5. Small molecular compounds**

| **Small Molecule** | **Description** | **Targets** | **Concentration(μM)** |
| --- | --- | --- | --- |
| Forskolin | activator of eukaryotic adenylyl cyclase (AC) | Adenylyl cyclase (AC) | 1.0 |
| SP600125 | JNK inhibitor | JNK1, JNK2, Aurora A, TrkA, JNK3 | 10.0 |
| PD 98, 059 | MEK inhibitor | MEK1 | 10.0 |
| GSK-LSD1 2HCl | LSD1 inhibitor | LSD1 | 0.5 |
| AG-490 | EGFR inhibitor | EGFR | 20.0 |
| IFN-γ | Cytokine | IFN-γ receptor | 20.0 ng/ml |
| BAY 11-7082 | NF-κB inhibitor | E2-conjugating enzymes, IκBα phosphorylation | 3.0 |
| GSK126 | EZH2 methyltransferase inhibitor | EZH2 | 1.0 |
| LY294002 | PI3Kα/δ/β inhibitor | P110α, p110δ, p110β, DNA-PK | 10.0 |
| Paclitacel | microtubule polymer stabilizer | Microtubule (human endothelial cells) | 0.1 |
| Etoposide | DNA synthesis inhibitor | Topo II | 0.1 |
| JQ1 | Bromodomains | BRD2, BRD3, BRD4, BRDT(BET) | 0.1 |
| Fludarabine | STAT1 activation inhibitor | STAT1 | 0.5 |
| Oltipraz | Nrf2 activator | Nrf2 | 10.0 |
| Selumetinib | MEK inhibitor | MEK1, MEK2, MEK3 | 0.1 |
| 5’AZA | DNA methyltransferase activity inhibitor | DNMTs | 1.0 |
| ATRA | the retinoic acid receptor (RAR) and the retinoid X receptor (RXR) ligands | RAR, RAX | 1.0 |

**
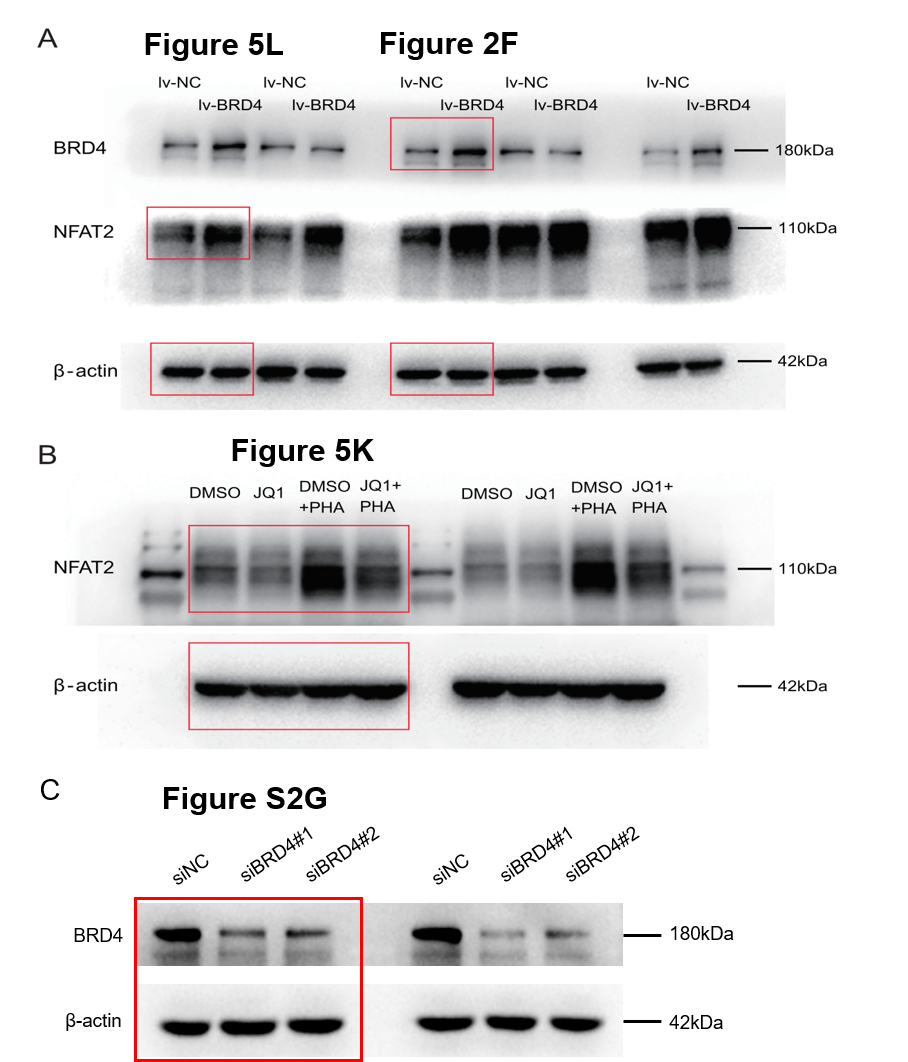
**

**Supplemental Figure 1.** Gel images for those cropped in the paper figures.

The specific bands shown in Figure 2F are highlighted by the red box on the right of (A). The specific bands shown in the Figures 5K and 5L are highlighted by red boxes in (B) and the left of (A) respectively. The specific bands shown in Figure S2G are highlighted by the red box in (C).


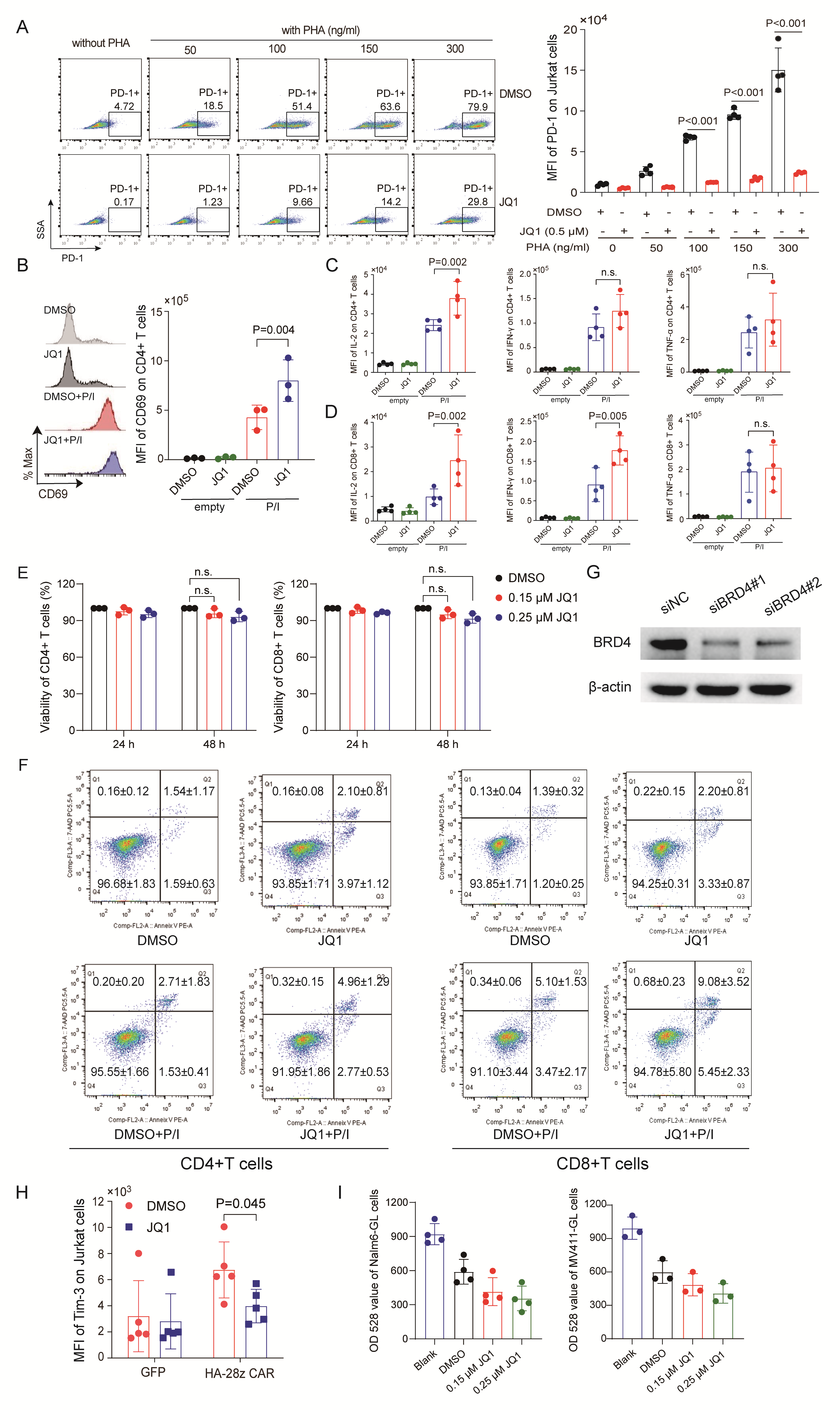


**Supplemental Figure 2.** JQ1 suppresses PD-1 expression of Jurkat cells and T cells, and promotes T cells function without inducing apoptosis or affecting viability, related to Figure 1-3.

(A) Jurkat cells were treated with 0.5 μM JQ1 and the indicated doses of PHA for 24 hours, and PD-1 expression was determined by FACS (*n* = 4). (B) CD4+ T cells from healthy donors were treated with 0.5 μM JQ1 for 24 hours in the presence and absence of P/I (PMA: 20 ng/L, ionomycin: 0.5 μM), and CD69 expression was determined by FACS (*n* = 3). (C) CD4+ T cells were treated with 0.5 μM JQ1 for 24 hours, and the secretions of IL-2 (left), IFN-γ (middle) and TNF-α (right) were evaluated by intracellular flow cytometry (*n* = 4). (D) CD8+ T cells were treated with 0.5 μM JQ1 for 24 hours, and the secretions of IL-2 (left), IFN-γ (middle) and TNF-α (right) were detected by intracellular flow cytometry (*n* = 4). (E) CD4+ T cells (left) and CD8+ T cells (right) were treated with 0.15 μM and 0.25 μM JQ1 for 24-48 hours, and the proliferation was determined by CCK8 (*n* = 3). (F) CD4+ T cells (left) and CD8+ T cells (right) from healthy donors were treated with 0.5 μM JQ1 for 24 hours in the presence and absence of P/I (PMA: 20 ng/L, ionomycin: 0.5 μM), and apoptosis was determined by FACS (*n* = 3). (G) The expression of BRD4 of Jurkat cells after BRD4 knockdown by siRNA was examined by western blot. (H) HA-28z CAR Jurkat T cells were treated with 0.5 μM JQ1 for 24 hours, and Tim-3 expression was determined by FACS (*n* = 5). (I) After being administrated by the indicated doses of JQ1 for 72 hours, CD19-CAR T cells or CD123-CAR T cells were co-cultured with Nalm6-GL cells or MV411-GL cells for 48 hours, and the 528nm OD value of Nalm6-GL cells (*n* = 4) or MV411-GL cells (*n* = 3) was detected by microplate reader. Data were expressed as mean ± SD. *n* = 3 or more independent biological replicates, presented as individual points. *P* value < 0.05 was considered to be significant (one-way ANOVA with Bonferroni post-hoc test).


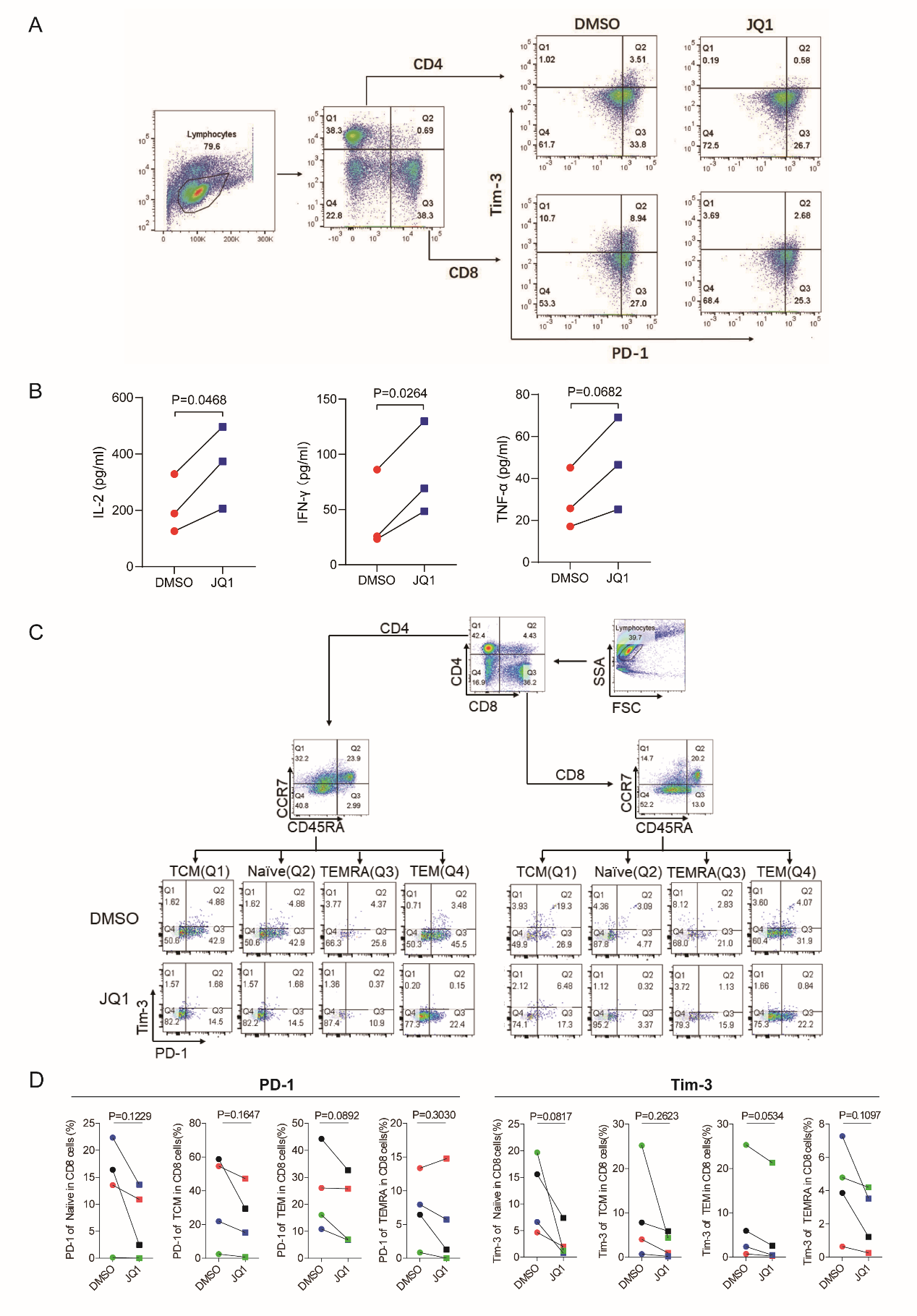


**Supplemental Figure 3.** Gating strategies for flow cytometry, and BET inhibitions reduce PD-1 and Tim-3 expression in subsets of T cells from patients, related to Figure 4.

(A) Representative flow cytometric analysis of the difference in PD-1 and Tim-3 expression between the DMSO and JQ1 groups in one of the samples. (B) CD3+ T cells from newly diagnosed AML patients were treated with 0.5 μM JQ1 for 24 hours, and the secretions of IL-2, IFN-γ, and TNF-α of CD3+ T cells were evaluated by ELISA (*n* = 3). (C) Representative flow cytometric analysis for one of the AML samples for PD-1 and Tim-3 expression in subsets of CD4+ and CD8+ T cells after JQ1 treatment. (D) CD3+ T cells from AML patients (*n* = 4) were treated with 0.5 μM JQ1 for 24 hours, and the percentages of PD-1 positive (upper row) and Tim-3 positive (lower row) cells in naive (left), TCM (second column), TEM (third column), and TEMRA (right) CD8+ T cells were determined by FACS. Data were expressed as mean ± SD. *n* = 3 or more independent biological replicates, presented as individual points. *P* value < 0.05 was considered to be significant (two-tailed paired Student’s t-tests).


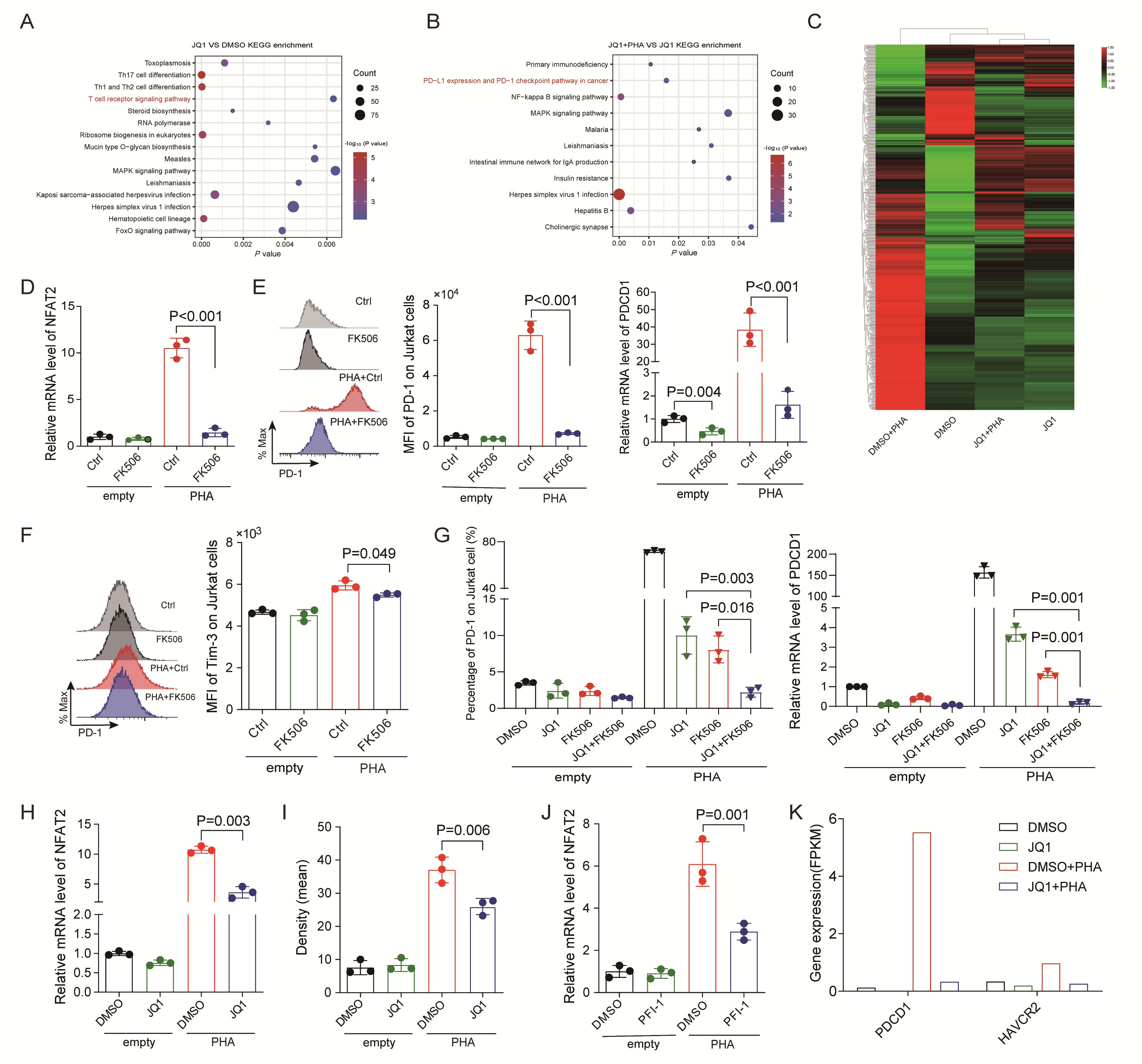


**Supplemental Figure 4.** BRD4 inhibition rescues T cell exhaustion via the NFAT2 signaling pathway, related to Figure 5.

(A) The top 15 enriched terms of the KEGG enrichment pathway analyses were shown in the bubble plot (DMSO VS JQ1). (B) Scatter plot of top 11 enriched KEGG pathways were shown in the bubble plot (JQ1 VS JQ1+ PHA). (C) Heatmap of the differential expression of genes in Jurkat cells was analyzed by RNA-seq. (D) Jurkat cells were cultured with 100 ng/ml FK506 for 24 hours with and without 150 ng/ml PHA stimulation, NFAT2 expression was determined by qRT-PCR (*n* = 3). (E) Jurkat cells were cultured with 100 ng/ml FK506 for 24 hours with and without 150 ng/ml PHA stimulation, PD-1 expression was determined by FACS (left) and qRT-PCR (right) (*n* = 3). (F) Jurkat cells were cultured with 100 ng/ml FK506 for 24 hours with and without 150 ng/ml PHA stimulation, Tim-3 expression was determined by FACS (*n* = 3). (G) Jurkat cells were treated with 0.5 μM JQ1 or/and 100 ng/ml FK506 in the presence and absence of PHA (150 ng/mL), and the expression of PD-1 was detected by FACS (left) and qRT-PCR (right) (*n* = 3). (H) Jurkat cells were treated with 0.5 μM JQ1 for 24 hours in the presence and absence of PHA (150 ng/mL), NFAT2 expression was determined by qRT-PCR (*n* = 3). (I) Quantitative analysis of intra nuclear immunofluorescence of each group. (J) Jurkat cells were cultured with 5 μM PFI-1 for 24 hours with and without PHA (150 ng/L) stimulation, and NFAT2 expression was determined by qRT-PCR (*n* = 3). (K) After Jurkat cells were treated with 0.5 μM JQ1 for 24 hours in the presence and absence of PHA (150 ng/mL), the expression of PDCD1 and HAVCR2 (FPKM) were analyzed by RNA-seq analysis. Data were expressed as mean ± SD. *n* = 3 or more independent biological replicates, presented as individual points. *P* value < 0.05 was considered to be significant (one-way ANOVA with Bonferroni post-hoc test).


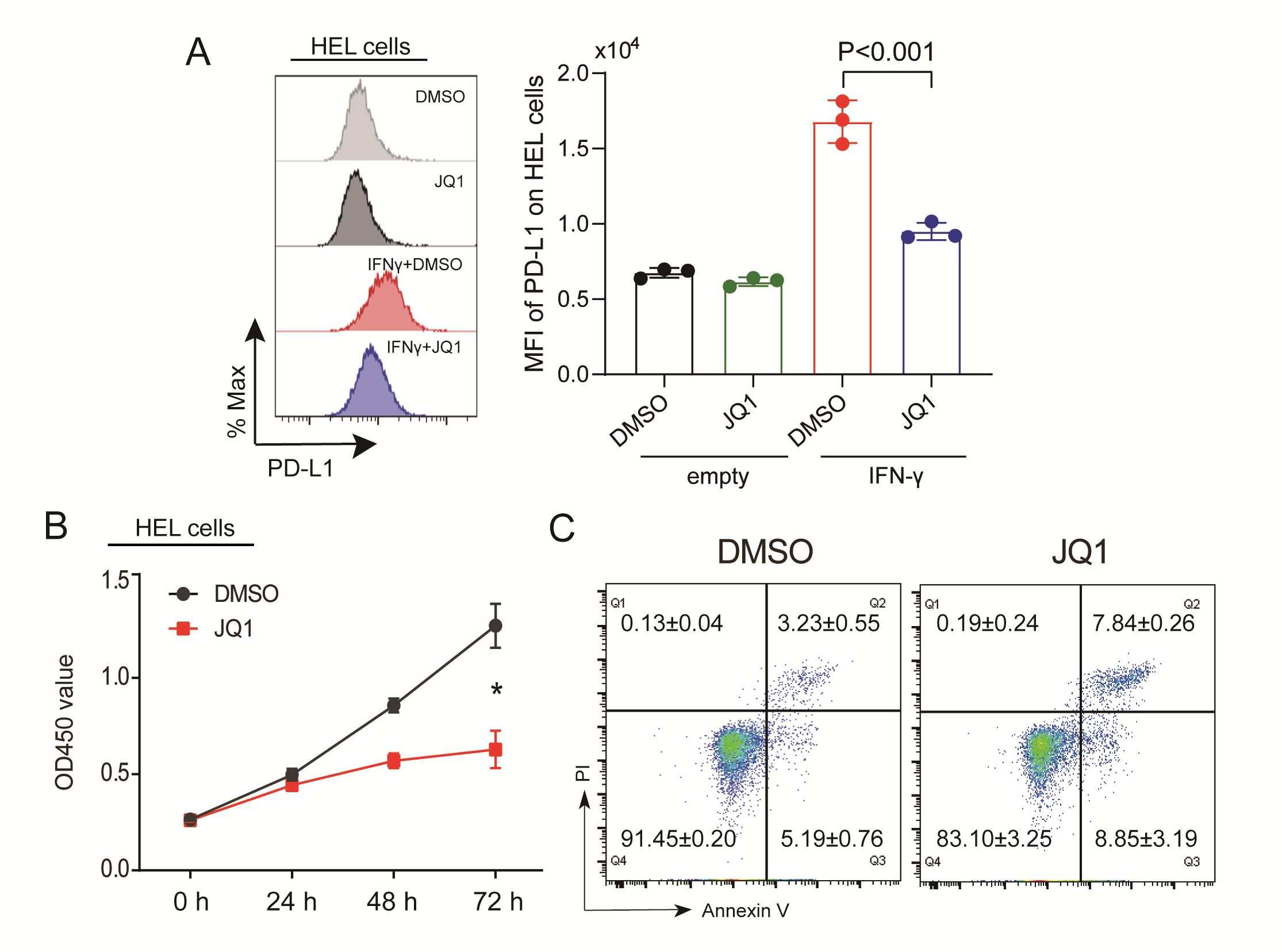


**Supplemental Figure 5.** BET inhibitor suppresses PD-L1 expression and cell growth, and promotes cell apoptosis in AML, related to Figure 6.

(A) HEL cells were treated with 0.5 μM JQ1 for 24 hours in the absence and presence of 20 ng/ml IFN-γ, PD-L1 expression was determined by FACS (*n* = 3). (B) HEL cells were treated with 0.5 μM JQ1 for 24 hours in the absence and presence of 20 ng/ml IFN-γ, then the proliferation was evaluated by CCK-8 assay at the indicated time point (*n* = 3). (C) HEL cells were treated with 0.5 μM JQ1 for 24 hours in the absence and presence of 20 ng/ml IFN-γ, apoptosis was determined by FACS (*n* = 3). Data were expressed as mean ± SD. *n* = 3 or more independent biological replicates, presented as individual points. *P* value < 0.05 was considered to be significant (one-way ANOVA with Bonferroni post-hoc test).


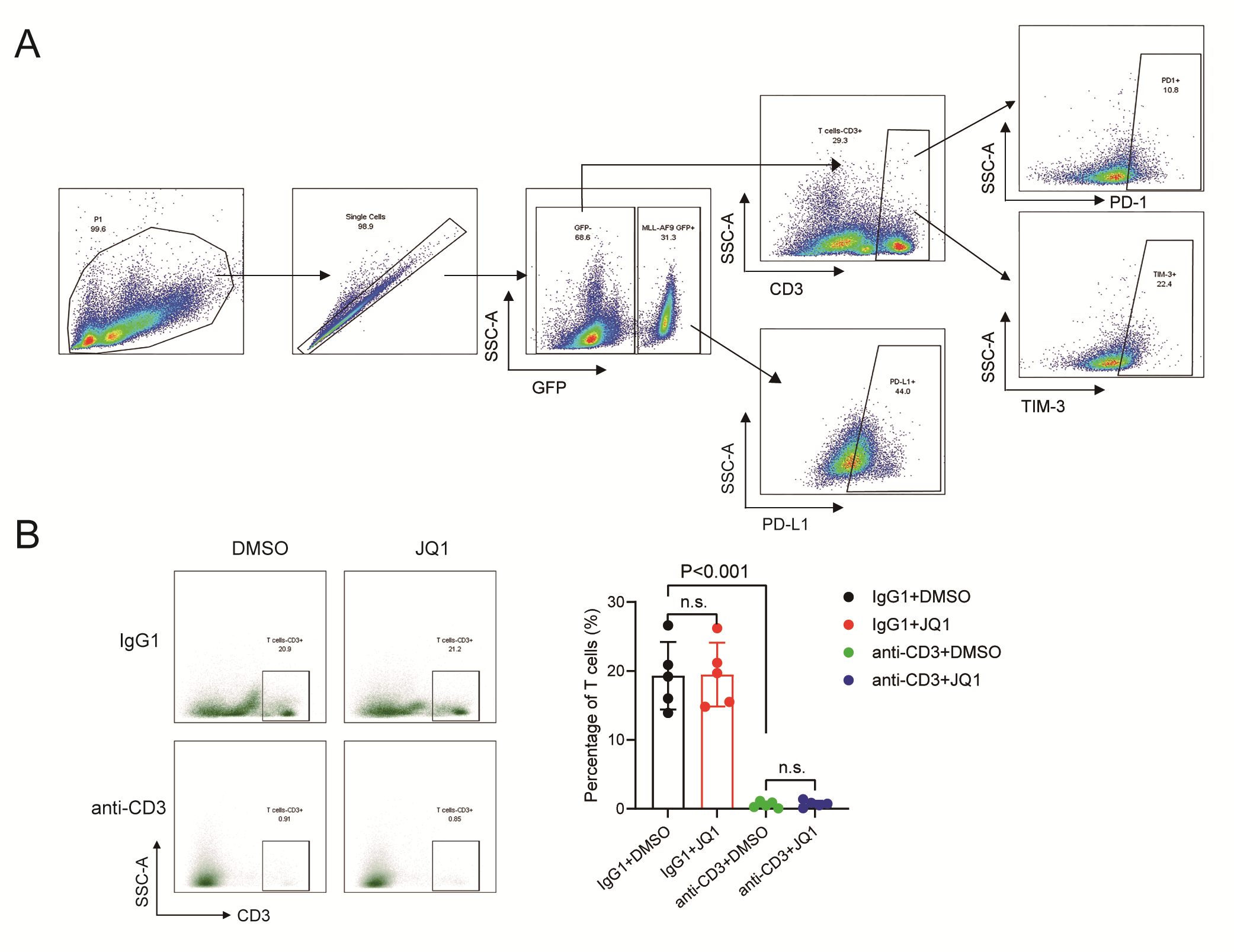


**Supplemental Figure 6.** Gating strategies for flow cytometry, and the percentage of T cells in the circulation of C57 mice treated with IgG1 or anti-CD3.

(A) Representative flow cytometric analysis for one of the MLL-AF9-bearing mice for the expression of CD3, PD-1, Tim-3 and PD-L1. (B) The percentage of T cells in the circulation of C57 mice treated with the indicated administration (*n* = 5 mice per group, one-way ANOVA with Bonferroni post-hoc test).
