# Supplementary material for: BET bromodomain inhibition rescues PD-1-mediated T-cell exhaustion in acute myeloid leukemia
Source: Cell Death Dis. 2022 Aug 2;13(8):671. doi: 10.1038/s41419-022-05123-x (PMC9346138; doi:10.1038/s41419-022-05123-x)
Supplement: Supplementary file 3 — Full length western blots [file 41419_2022_5123_MOESM3_ESM.doc]

**
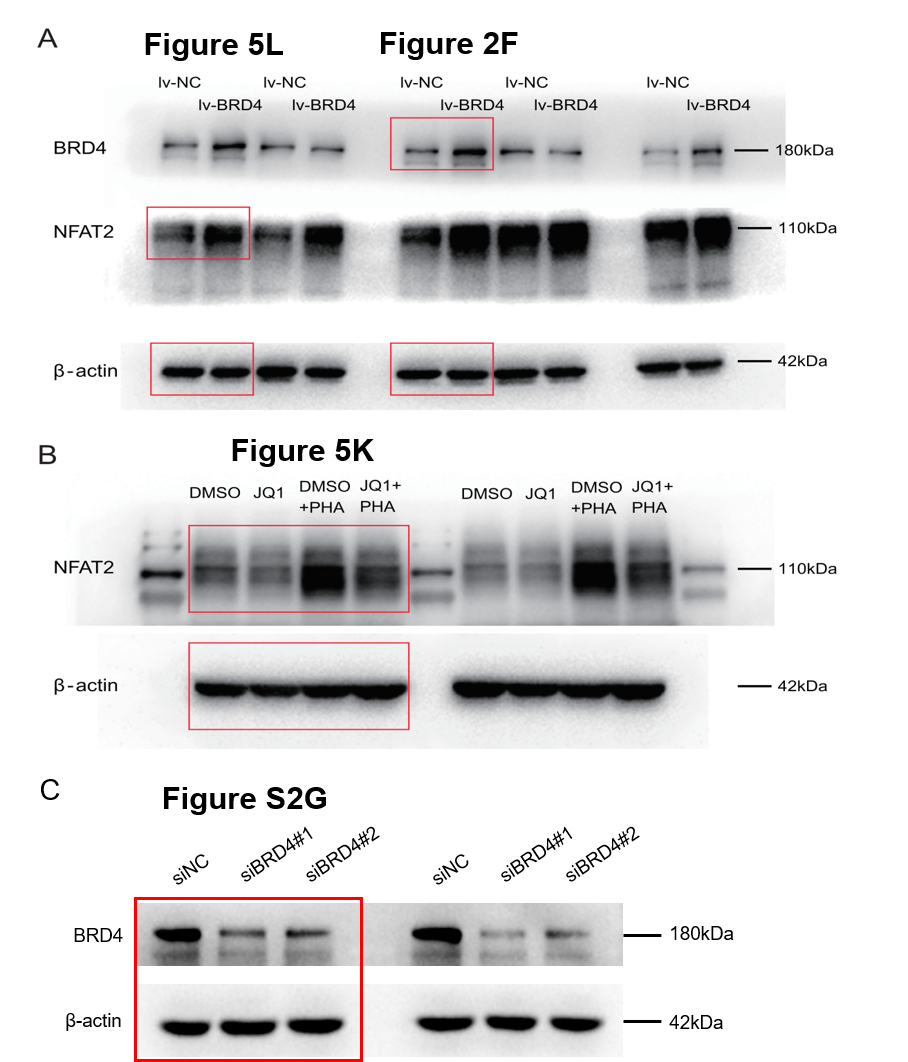
**

**Supplemental Figure 1.** Gel images for those cropped in the paper figures.

The specific bands shown in Figure 2F are highlighted by the red box on the right of (A). The specific bands shown in the Figures 5K and 5L are highlighted by red boxes in (B) and the left of (A) respectively. The specific bands shown in Figure S2G are highlighted by the red box in (C).
